# Oxidative Stability of Walnut Kernel and Oil: Chemical Compositions and Sensory Aroma Compounds

**DOI:** 10.3390/foods11193151

**Published:** 2022-10-10

**Authors:** Josephine Ampofo, Filipa S. Grilo, Sue Langstaff, Selina C. Wang

**Affiliations:** 1 Department of Food Science and Technology, University of California Davis, Davis, CA 95616, USA; 2 Applied Sensory, Fairfield, CA 94534, USA

**Keywords:** oxidation, walnuts, sensory attributes, volatiles, rancidity, antioxidants

## Abstract

The impact of storage temperature and time on quality of two walnut cultivars (*Juglans regia* Chandler and Howard) were evaluated. Free fatty acids, peroxides, and oxidative stabilities exhibited significant changes. After the storage period, γ-, δ-, and α-tocopherols in Howard oil significantly reduced by 42, 56, and 100% at 5 °C, while 23 °C showed 48, 42, and 100% losses, respectively. For Chandler oil, storage at 5 °C reduced γ-, δ-, and α-tocopherols by 19, 24, and 100%, while 23 °C caused 42, 45, and 100% losses, respectively. Storage of Howard kernels, up to month four, significantly reduced total phenolics by 9 and 18%, at 23 and 5 °C, respectively, whereas Chandler also reduced by 9 and 27%, at 23 and 5 °C, respectively. Additionally, 14 phenolic compounds were profiled in kernels, where flavonoids were dominant than phenolic acids. At the end of month four, the dominant phenolic compound was gallic acid at 23 °C (981.68 and 703 mg/kg for Chandler and Howard, respectively). Additionally, positive correlations were observed between rancid sensory perceptions and oxidative volatiles. Storage conditions are crucial for maintaining the sensory and nutritional attributes of walnuts during postharvest management.

## 1. Introduction

Global production of walnut (*Juglans regia* L.) has increased from 2.96 million to 3.32 million metric tonnes from 2017 to 2020, with China, United States, and Iran being the key producers [1]. Consumption of walnuts has been practiced since ancient times, due to their unique organoleptic qualities and health benefits, such as anti-cardiovascular, -cholesterol, and -oxidative effects [2,3]. The sensory and health attributes of walnuts are associated with compounds such as phenolics, tocopherols, squalene, and unsaturated fatty acids [4,5].

Walnuts are consumed in their whole kernel forms or as ingredients in foods, such as cheese, baked goods, yoghurt, candy, chocolate, snack bars, and smoothies [2]. A key factor that contributes to applications of walnut in the food industry is shelf-life stability, which is the length of time required for a food to remain fit for consumption or sale [6]. Apart from genetic influence, the shelf-life stability of walnuts is determined by undesirable oxidative changes, due to their exposure to temperature, light, moisture, relative humidity, and atmospheric oxygen during postharvest handling [7]. Previous studies have linked kernel oxidation to their high fat content (52 to 70 g/100 g, depending on cultivar), mainly composed of unsaturated fatty acids, such as linoleic (49.3–62.3 g/100 g), oleic (13.8–33.0 g/100 g), and linolenic (8.0–15.4 g/100 g) acids [8,9]. According to Phatanayindee [10], the high susceptibility of unsaturated fatty acids to free-radical chain reactions in the presence of heat and oxygen leads to the formation of secondary products (e.g., hydroperoxides, malonaldehyde, and ketones) responsible for oxidative rancidity, discoloration, and poor nutritional value. For instance, a recent study from our group established positive correlations between storage conditions, oxygen availability, oxidative stability, and rancidity in four walnut cultivars [11]. Additionally, Jensen et al. [12] investigated walnut oxidative rancidity under different storage conditions (i.e., light/darkness and 5/21 °C) for 25 weeks. From their results, storage under light at 21 °C had the highest oxidative rancidity, compared to 5 °C, which further showed no trace of oxidative rancidity, even under darkness.

Despite these reports, the literature regarding the minimum intensity levels at which chemical oxidative markers are correlated with walnut quality and sensory acceptance is very scanty. Since the production of chemical oxidative markers are directly linked with postharvest management practices, there is the need to investigate and report how factors such as storage temperature and time influence walnut quality along the postharvest chain. Additionally, the literature regarding how different fractions of walnut (i.e., kernel and oil) respond to storage conditions for quality deterioration is limited. Thus, this study investigated the impact of different storage factors (i.e., temperature and time) on the accumulation of chemical oxidative markers in different fractions (kernel and oil) of two popularly grown California walnut cultivars (i.e., Chandler and Howard)**.** Additionally, this study explored the relationship between the profiled oxidative markers and sensory perception of rancidity in walnut along the storage period.

## 2. Materials and Methods

### 2.1. Walnut Samples

Fresh kernels of two walnut cultivars (*Juglans regia*—‘Chandler’ and ‘Howard’) were harvested at full maturity stage in September; we obtained them from a grower and processor in California.

### 2.2. Storage Experiment

Each walnut cultivar (18 kg) was stored in opened transparent containers inside a temperature-controlled chamber at 50% RH under florescent light. Samples were stored at 5 and 23 °C for a total of 4 months. At the end of each storage month, kernels (1 kg) from each temperature condition were collected for chemical and sensory analyses. Kernel oil extraction was conducted within 3 h after each sampling period.

### 2.3. Quality Parameters

#### 2.3.1. Kernel Moisture Analysis

Moisture content was evaluated as described by Grilo and Wang [13]. Approximately 40 g of each investigated kernel was milled with a food processor (Model DLC-2A; Cuisinart Mini-Prep Plus^®^; East Windsor, NJ, USA), transferred into a 600 mL beaker, and then dried in an oven at 105 °C, until a constant weight was reached. Afterwards, dried samples were kept in a desiccator to reach room temperature and then reweighed.

#### 2.3.2. Oil Extraction

Oil from dried kernels was extracted according to the method of Grilo and Wang [13]. Walnut kernels were pressed with an electrical resistance-heating ring attached around the press barrel at 27 °C. Subsequently, oil from the pressed kernels was extracted with a screw press and KK oil Prince F universal (Reut, Germany), consisting of a 7 mm restriction die and screw speed of 20 rpm. Prior to extraction, the equipment was operated for 10 min to reach its optimal extraction temperature (30 °C) without the presence of kernels.

#### 2.3.3. Evaluation of Peroxide Value, UV Absorbances, Oxidative Stability and Free Fatty Acids

Changes in peroxide value (PV), UV absorbances (K_232_ and K_268_), oxidative stability, and free fatty acids were estimated as described by Grilo and Wang [13]. Standard methods of American Oil Chemistry Society (2009), Cd 8b-90(09), and Ch 5-91(09) were employed to determine oil PV, K_232_, and K_268_, respectively. Oxidative stabilities of kernel and oil were measured with a Rancimat apparatus 617 (Metrohm AG, Herisau, Switzerland). In summary, 3 g of oil and 0.5 g of milled kernel samples were oxidized at 110 °C, with 20 l/h of airflow.

### 2.4. Profiling of Oil Fatty Acids

Fatty acid composition of each extracted oil was evaluated by gas chromatography/mass spectrometry, as previously described by Grilo and Wang [13]. Briefly, 0.1 g of sample aliquots were diluted in 1 mL of n-hexane and vortexed for 10 s. Afterwards, the obtained mixture was mixed with 0.1 mL of 2N KOH in methanol and vortexed for 2 min. Subsequently, 500 µL of the transparent organic phase containing fatty acid methyl esters was decanted, diluted to a final volume of 1 mL with n-hexane, and then analyzed within 12 h. Profiling of fatty acids was conducted with a gas chromatograph (7890A, Agilent Technologies, Palo Alto, CA, USA) equipped with a split injector and flame ionization detector equipped with a capillary column ZB-23 (20 m, 180 µm, and 0.2 µm). A column initial temperature of 80 °C was employed for 0.5 min, then increased to 175 °C at a rate of 65 °C/min, and, finally, to 230 °C at a rate of 7 °C/min. Temperatures were held at 0, 0.5, and 5 min, respectively, at each stage of programming. The injector and detector were held at 250 and 260 °C, respectively, with an injection volume of 1 µL. Fatty acid methyl esters were identified using a mix of 37-component fatty acid methyl esters purchased from Supelco (Sigma-Aldrich, St. Louis, MO, USA). Quantification of fatty acids was performed using an Agilent Open Lab ChemStation for Windows.

### 2.5. Extraction and Analysis of Oil Tocopherols

Tocopherols were extracted as outlined by Gimeno et al. [14] and modified by Grilo and Wang [13]. Forty microliters of walnut oil were vortexed with 160 µL of hexane. Afterwards, 600 µL of methanol and 200 µL of an internal standard solution (α-tocopherol acetate in ethanol, 300 µg/mL) were added, vortexed for 1 min, and centrifuged (1788.8× *g*, 5 min, 23 °C). Subsequently, the obtained oil mixture was stored at −20 °C for 1 h to allow for separation of oil from the organic phase of the mixture. After this, the organic extract was filtered with 0.45 µm (nylon) and analyzed using UPLC-DAD. Agilent 1290 Infinity II LC system with a diode-array detector and Agilent ZORBAX Eclipse Plus C18 column (3.5 µm, 3 × 100 mm) were used to conduct the analysis, with a mobile phase of methanol:water (96:4), injection volume of 20 µL, and flow rate of 1.0 mL/min. The DAD signal was recorded at 292 nm, after each run time of 12 min. Identification of tocopherols were conducted by comparing their retention time to their respective pure standards (δ-, γ-, and α-tocopherols).

### 2.6. Kernel Total Phenolic Content

Total phenolic content (TPC) of investigated kernels was evaluated according to the method of Ojeda-Amador et al. [15]. Briefly, 0.25 g of grounded kernel was mixed with 5 mL of methanol/water/formic acid (80/19/1, *v*/*v*/*v*), vortexed (2 min), sonicated (5 min), and centrifuged (5000 rpm, 10 min) subsequently. Obtained supernatant was stored in the dark, and re-extraction from pellets was repeated twice. Afterwards, all three obtained supernatants were combined, and methanol was evaporated with a vacuum evaporator at 30 °C. Dried extracts were redissolved in 1 mL methanol prior to analysis. TPC was quantified by the Folin–Ciocalteu method, using a gallic acid calibration curve (ranges 10.1–504.6 µg/mL) [16]. Results were expressed as mg/kg of gallic acid in kernel.

### 2.7. Profiling of Kernel Phenolic Compounds

Redissolved phenolic extracts were membrane-filtered with 0.45 µm cellulose filter before HPLC-DAD analysis, as described by Ersan et al. [17]. HPLC-DAD model (Agilent G4212-60008, serial number—DEBAF01604) was used, with the following solvents: HPLC grade water (99%, Solvent A) and HPLC grade methanol (99%, Solvent B), with each solvent containing 1% (*v*/*v*) formic acid. The column was an Eclipse Plus C18 column (250 mm × 4.6 mm, 5 µm; Agilent, USA), with a security guard ultra C18 guard column (4.6 mm × 2 mm) of the same material. The gradient included: isocratic at 2% B for 10 min, then 2 to 37% B in 27 min, isocratic at 37% B for 5 min, then from 37 to 40% B in 18 min, from 40 to 60% B in 10 min, from 60 to 100% B in 20 min, isocratic at 100% B for 14 min, then from 100 to 2% B in 1 min, and isocratic at 2% B for 7 min. Column temperature was 35 °C, and the total run time was 112 min at a flow rate of 1 mL/min and injection volume of 3 µL. The UV/vis absorption spectra were recorded at 280 nm (gallactotannins), 310 nm (anacardic acids), and 350 nm (flavonols). Results were expressed as mg/kg of kernel or oil.

### 2.8. Characterization of Kernel and Oil Volatile Compounds

Volatile compounds from kernel and oil were extracted by solid-phase microextraction (SPME) [13]. Briefly, 3.0 g of grounded kernel flour or extracted oil was mixed with 2.5 mg/kg of 4-methyl-2-pentanol (internal standard), stirred for 10 min at 40 °C until equilibration in a 20 mL glass vial with a PTFE/silicone septum (Agilent Technologies, Palo alto, CA, USA). Next, a solid phase microextraction (SPME) fiber (DVB/CAR/PDMS, Sigma-Aldrich, St. Louis, MO, USA) was exposed to the sample headspace for 40 min. Next, the volatile compounds were analyzed with a GC system (Agilent Technologies) comprising an autosampler (Agilent PAL RSI 85), gas chromatograph (GC Agilent 7820A) and mass spectrometer (Agilent 5977B) designed with an electron impact source and quadrupole analyzer. Volatile compounds were separated with a Supelcowax 10 column (30 m × 0.25 mm × 0.25 µm, Sigma-Aldrich, USA) using helium as a carrier gas at a flow rate of 1 mL/min. GC oven temperature was started at 40 °C and increased by 3 °C/min after 10 min, until it reached a final temperature of 200 °C. Volatiles were identified by two methods, including the use of NIST 08 Mass Spectral Library and comparison of their retention time and mass spectrum to their respective standards. Results were expressed as µg of internal standard per kg of kernel or oil sample.

### 2.9. Sensory Analysis

Both walnut kernel and oil samples obtained from each storage condition were evaluated using the objective sensory method of descriptive analysis. The panelists selected for this project had many years of experience using this methodology for evaluating food and beverage products. The panelists (i.e., 2 male and 6 female; all above 30 years.) participated in a two 2.5 h sessions to develop sensory terminology to describe the walnut kernels and the walnut oils. After reaching a consensus, the panelists selected seven attributes for the walnut kernels and fifteen for the walnut oils. The descriptive terms for the kernels were: honey aroma, cardboard aroma, rancid aroma, crunchy, rancid flavor, bitterness and astringency/drying. The fifteen attributes for the walnut oils comprised seven aroma and eight flavor attributes: overall aroma intensity, buttery aroma, fresh nuts aroma, toasted/burnt aroma, grainy aroma, cardboard/woody aroma, rancid aroma, overall flavor intensity, sweetness, fresh nuts flavor, toasted flavor, grainy flavor, cardboard flavor, rancid flavor and greasy/thick texture. 

For the walnut kernels, eleven samples plus one control (month 0) were evaluated over two sessions: Chandler on one day and Howard on a separate day. A modified Latin square design was used each day to randomize the presentation of the twelve samples among the eight panelists.

Kernels were served in clear, tulip-shaped wine glasses of 220-mL capacity which were coded with 3-digit random numbers. Two kernel halves were placed in each glass and then covered with a 5.7 cm diameter plastic Petri dish for at least 2 h prior to evaluation. The tests were conducted in a room illuminated with “daylight” fluorescent lighting. All samples were served at room temperature (20–23 °C). The panelists were separated by dividers and were not allowed to communicate during the session. Panelists expectorated the samples and rinsed with bottled water between tastings. Red seedless grapes were also available as a palate cleanser. Each judge rated the intensity of the attributes for the different samples using structured 10-point (0 to 10) scales anchored at the ends with terms “low” and “high” or “smooth” and “grippy.” Previously agreed upon reference standards were available in each booth. Similarly, the eleven walnut oils samples, in addition to a control, were evaluated over two sessions: Chandler on one day and Howard on a separate day. A modified Latin square design was used each day to randomize the presentation of the twelve samples among the eight panelists.

The oils were served in clear, tulip-shaped plastic wine glasses of 133-mL capacity which were coded with 3-digit random number codes. Approximately 12 mL of oil were poured in each glass and then covered with a 4.5 cm diameter plastic cover for one hour prior to evaluation. The plastic cups with the oils were put on metal trays and the trays were placed on heating mats in each sensory booth. All samples were served at a temperature of 28 ± 2 °C. The tests were conducted in a room illuminated with “daylight” fluorescent lighting. The panelists were separated by dividers and were not allowed to communicate during the session. Panelists expectorated the samples and rinsed with bottled water between tastings. Each judge rated the intensity of the attributes for the different samples using structured 10-point (0 to 10) scales anchored at the ends with terms “weak” and “strong,” or “low” and “high.” Previously agreed upon reference standards were available in each booth. 

### 2.10. Data Analysis

All chemical analyses described in this study were conducted in triplicates. Statistical analyses were performed using SPSS software (SPSS Inc., Chicago, IL, USA). Biochemical markers and sensory attributes were analyzed using a two-factorial model (time and temperature) at five (0, 1, 2, 3, and 4 months) and two (5 and 23 °C) levels, respectively. Significant differences were tested by Tukey’s multiple comparison test at *p* ≤ 0.05. Correlation analysis between oxidative volatiles and sensory attributes was performed by Pearson’s correlation test. XLSTAT (version 2013) was used for partial least squares regression analysis to evaluate the relationship between chemical oxidative markers and sensory perception of rancidity.

## 3. Results and Discussion

### 3.1. Impact of Storage on Quality Parameters

#### 3.1.1. Moisture

Postharvest storage conditions influence food quality by inducing undesirable changes in biochemical make-up [18]. As depicted in Table 1, significant differences (*p* < 0.05) in quality parameters were observed during walnut storage. For moisture content, Howard kernels recorded the highest value (7.88 g/kg), with 5 °C/month 4, with this observation being significantly higher by 34, 52, and 47.59%, compared to their respective 23 °C (i.e., at the same storage time) and raw samples. Similar to Howard, the moisture content for Chandler kernels showed an increasing trend, with 5 °C along the storage period. Overall, the Chandler moisture content at 5 °C/month 4 was 1.7 and 1.75 times higher than with 23 °C (i.e., at the same storage time) and the raw kernel samples, respectively. The high moisture content observed with 5 °C can be attributed to the high humidity at this temperature, which resulted in the absorption of water by the kernels. This observation is crucial because it can lead to early deterioration and loss of quality.

#### 3.1.2. Free Fatty Acids and Peroxide Value

Production of free fatty acids (FFA) is due to the hydrolysis of triacylglycerols through hydrothermal conditions or the action of lipases [19]. FFA results from this study showed a simultaneous trend for both Howard and Chandler, where FFA did not show significant changes from month 1 up to month 3. However, prolongment of storage period up to month 4 significantly increased (*p* < 0.05) the FFA by 33.33% at both 5 and 23 °C. Irrespective of cultivar type, the low FFA observed with raw samples may be linked with the low moisture contents of their kernels, compared to their respective stored kernels. This may have limited lipase activity for FFA formation [20]. Additionally, the significant increment of FFA at month 4 for all investigated temperatures signifies the potential activation of kernel matrix breakdown with the prolongment of storage period. This result confirms our previous storage study on oils from different walnut cultivars [13].

Peroxide value (PV) is an indicator of the formation of hydroperoxides as primary oxidative products [21]. As expected, PV increased significantly (*p* < 0.05) along storage. PV from Howard showed the highest value of 1.80 meq O_2_/kg at month 4 for both 5 and 23 °C, with this observation being 52.78 and 48.33% higher, compared to their respective controls. For Chandler, although PV at month 1 was significantly higher at 5 °C (1.06 meq O_2_/kg), compared to 23 °C (0.93 meq O_2_/kg), prolonging storage time up to month 4 increased PV to 1.80 meq O_2_/kg at 23 °C. According to Buransompob et al. [22] high quality walnut should record a PV limit of 3.0 meq O_2_/kg. Thus, it can be postulated that, storage conditions investigated in this study are appropriate to enhance walnut shelf-life stability during postharvest. Interestingly, a current work published by our research group observed higher PV ranges for Chandler (1.0–4.3 meq O_2_/kg) and Howard (1.8–6.4 meq O_2_/kg) between 1 to 28 weeks of storage at 25 °C [13]. Thereby providing a clear indication of the impact of different storage conditions on walnut rancidity [13].

#### 3.1.3. UV Absorbances

UV absorption at 232 (K_232_) is associated with the presence of hydroperoxides, conjugated dienes, carboxylic compounds, and conjugated dienes, whereas absorption at 268 (K_268_) is a representation of the presence of secondary products formed from oxidative compounds discovered at K_232_ [21,23]. K_232_ values for Howard oils ranged from 0.90–2.50, with the peak value observed at 23 °C/month 4. This observation was significantly (*p* < 0.05) higher than observed with 5 °C/month 4 (by 38%) and control by 64%. Following a similar trend, higher levels of storage conditions (temperature and time) reported higher K_232_ values in Chandler oil, with 23 °C/month 4 showing the highest value of 1.94 than oil samples from 5 °C/month 4 (1.39) and control (1.06). Additionally, although K_268_ for Chandler oil at 5 °C was highest at month 1 (0.14), it showed a decreasing trend up to month 4, whereas its observation at 23 °C/month 4 showed a significant increment. For Howard oils, the highest value of K_268_ (0.18) was recorded at 23 °C/month 3 and 4. It should be highlighted that, the simultaneous synergy between increasing values of K_232_ and K_268_ at 23 °C/month 4 indicate a continuous oxidation under high storage temperature and time [24], which further explains the PV observations of this study. UV results of this study are lower than reported by Rabadán et al. [25], where the authors observed higher K_232_ in walnut (cv. Pedro) oil stored at 5 °C (i.e., 2.78) and 22 °C (i.e., 2.94).

#### 3.1.4. Oxidative Stability

Kernel oxidative stability at 5 °C ranged between 10.74–13.10 h for Howard and 12.07–13.97 h for Chandler, whereas, at 23 °C, it ranged between 11.07–14.07 h for Howard and 12.24–14.01 h for Chandler. Reduced kernel oxidative stabilities observed for both Howard and Chandler kernels under investigated storage temperatures may be associated, with the high moisture contents detected for both cultivars under the same storage conditions. Thus, confirming literature that high kernel moisture content leads to undesirable chemical changes capable of reducing oxidative stability [20]. Nevertheless, oil oxidative stability decreased with increasing storage period for both cultivars at 5 and 23 °C. For Howard oil, oxidative stability at 5 and 23 °C ranged between 2.51–2.84 h and 2.48–2.88 h, respectively. Chandler showed a similar trend of decreasing oil stability with increasing storage time, where the values observed at 5 and 23 °C ranged between 2.28–2.87 h and 2.03–2.72 h, respectively. In summary, storage at 23 °C reduced oil oxidative stability than at 5 °C, which is in synchrony with results observed for PV, K_232_, and K_268_.

### 3.2. Impact of Storage Conditions on Antioxidant Compounds

#### 3.2.1. Changes in Tocopherols

**Figure 1:**
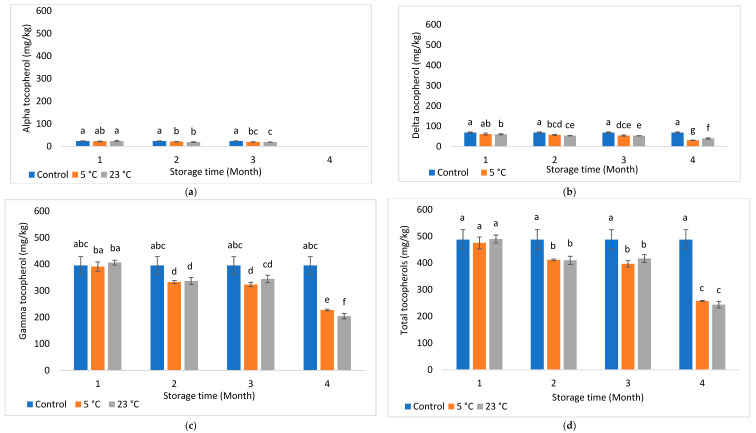
(**a**–**d**) Changes in alpha (**a**), delta (**b**), gamma (**c**), and total tocopherol (**d**) contents of Howard oil along storage. Different letters within indicate significant differences (*p* < 0.05) for storage time and month.

**Figure 2:**
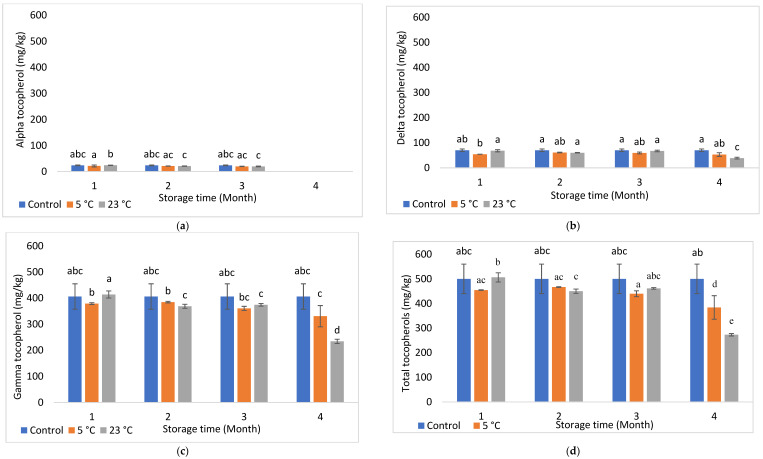
(**a**–**d**) Changes in alpha (**a**), delta (**b**), gamma (**c**), and total tocopherol (**d**) contents of Chandler oil along storage. Different letters within indicate significant differences (*p* < 0.05) for storage time and month.

The presence of antioxidant compounds in foods has been shown to help protect against oxidation by scavenging reactive oxygen and metal species [26]. Results for α-, δ-, γ, and total tocopherols are presented in Figure 1a–d (Howard oil) and Figure 2a–d (Chandler oil). Irrespective of cultivar or storage condition, γ-tocopherol was the most dominating among profiled tocopherols.

Furthermore, γ- and total tocopherols quantified for 5 °C Chandler oil were comparable to their control throughout the storage period, whereas, at 23 °C, they were comparable to the control, until month 4, where it reduced significantly (*p* < 0.05) by 45.47% (for total tocopherols) and 42.40% (for γ-tocopherol). Interestingly, α-tocopherol was absent at month 4 for both investigated temperatures irrespective of cultivar. This observation could be due to the preferential utilization of α-tocopherol against oxidative stress over other investigated tocopherols during storage. According to the literature, α-tocopherol is the most reactive and less stable form of tocopherol, followed by γ- and δ-tocopherols due to its lower bond dissociation energy required for abstraction of hydrogen from its hydroxyl group than other tocopherols [27]. Another trend with Chandler was the significant (*p* < 0.05) reductions of γ- (by 29.13%) and δ- (by 27.08%) tocopherols at 23 °C/month 4, compared to 5 °C, although 23 °C presented higher concentrations (i.e., 8.45 and 20.71% for γ- and δ-tocopherols, respectively, than 5 °C) at month 1. This trend was not the case for Howard, hence demonstrating the influence of genetics on tocopherol changes during postharvest handling. Additionally, higher tocopherol reductions at 23 °C indicate accelerated oxidation and the need to utilize available tocopherols as antioxidants.

#### 3.2.2. Changes in Kernel Phenolics Profile

Besides tocopherol, phenolic compounds are also linked with food quality and preservation. Basically, phenolic compounds are benzene ringed secondary metabolites containing hydroxyl groups [28]. In this study, 14 phenolic compounds were profiled in walnut kernels, with their concentrations being influenced by storage conditions (Table 2).

At the end of storage, total phenolic content of Howard reduced by 9 and 18% at 23 and 5 °C, respectively, compared to their concentrations prior to storage. For Chandler kernels, total phenolic content reduced by 26.93 and 28.04% at 23 and 5 °C, respectively, at the end of the storage period than its level prior to storage. Reductions in total phenolic content may be attributed to the presence of polyphenol oxidase (PPO), the main enzyme responsible for the oxidation of phenolic compounds into quinones for browning [29]. Phenolic compounds may also undergo non-enzymatic oxidation through redox cycles of Fe^3+^/Fe^2+^ and Cu^2+^/Cu^+^ [30]. However, the literature on enzymatic and non-enzymatic oxidation of walnut kernel phenolics is not fully elucidated. Similar to this study, Yildiz and Karaca [31] reported reduced total phenolic content in walnut kernels after 6 months of storage and attributed their findings to PPO activity. However, other previous studies also reported insignificant reductions in kernel total phenolic content along storage [32].

The 14 phenolic compounds identified in kernels were grouped into phenolic acids (i.e., ellagic, gallic, protocatechuic, vanillic, caffeic, *p*-coumaric, and chlorogenic acids), flavanols (i.e., epicatechin, gallocatechin gallate, gallocatechin, catechin, and epicatechin gallate), and flavonols (i.e., quercetin and rutin). Concentration of flavonoids (i.e., flavanols and flavonols) were more pronounced, compared to phenolic acids. The dominant phenolic compound for both Howard and Chandler was gallic acid, which was higher in Chandler (981.68 mg/kg) than Howard (703 mg/kg) at 23 °C/month 4. This observation was significantly higher (*p* < 0.05), compared to its concentration at 5 °C by 12.35% for Howard and 21.04% for Chandler, as well as its initial concentration prior to storage by 33.50% for Howard and 75.21% for Chandler. Besides gallic acid, other dominating phenolics included *p*-coumaric acid, caffeic acid, gallocatechin gallate, epicatechin gallate, quercetin, and rutin, with their concentrations dependent on storage temperature and time. At 23 °C/month 4, concentrations of gallocatechin gallate, rutin, *p*-coumaric acid, and caffeic acid were higher than at 5 °C by 10.76, 23.68, 33.89, and 51.69%, respectively. All other profiled phenolic compounds ranged between 17.34–20.74 mg/kg. The higher phenolic levels at 23 °C explained more oxidative stability at elevated temperatures. This result demonstrates the protective role of phenolics against oxidation by competing for free radicals and other endogenous pro-oxidants [33].

### 3.3. Effect on Walnut Oil Fatty Acid Profile

Investigated storage temperatures showed insignificant (*p* > 0.05) changes in unsaturated and saturated fatty acids. The previous literature has established the dense representation of unsaturated fatty acids in walnuts, among which, PUFAs are the most dominant [25,34,35]. In ascending order, linoleic acid (C18:2) was the most prepotent (59.61–60.69% for Howard and 60.25–60.81% for Chandler), followed by α-linolenic (C18:3; 15.54–15.87% for Howard and 14.52–14.88% for Chandler) and oleic acid (C18:1; 8.42–9.49% for Howard and 9.52–11.77% for Chandler) (Table 3). Results of this study suggests that the storage conditions investigated in this study were not only able to maintain PV values at acceptable levels but also protected concentrations of PUFAs. Contrary to our study, Christopoulos and Tsantili [36] reported significant monounsaturated reductions in Chandler kernels stored at 1 and 20 °C for 12 months. However, Descalzo et al. [37] observed no significant fatty acid reductions in pecan nuts stored at 2 and 20 °C for 0–10 months.

### 3.4. Kernel Volatile Changes

Upon exposure to heat and light, hydrogen atoms of the double bonds present in fat matrix of nuts are extracted to form alkyl radicals, which are further oxidized into hydroperoxides [18]. Hydroperoxides are subsequently broken down into carbonyl compounds (e.g., ketones, aldehydes, and alcohols), which have been shown to influence the flavor and quality of high lipid foods, such as walnuts [38]. The changes in oxidative volatiles of Howard and Chandler kernels are presented in Table 4 and Table 5, respectively.

Results of this study confirm the comment of Frankel [39], that is, that the low odor threshold of oxidative volatiles during storage leads to significant differences in volatile profile compared to fatty acids. Overall, 25 volatiles belonging to aldehydes, alcohols, esters, furans, ketones, and acids were profiled and exhibited significant changes, based on storage time and temperature. At the end of the storage period, although the highest level of hexanal (1238.37 mg/kg), heptanal (9.32 mg/kg), *E*-2-hexenal (7.50 mg/kg), and *E*-2-heptenal (13.60 mg/kg) were observed at 5 °C, and they were not significantly different from their respective results at 23 °C for Howard. Thus, it can be inferred that the evolution of the above-mentioned volatiles in Howard kernels was independent of the storage temperature, but dependent on storage time. Additionally, all six profiled alcohols in Howard kernel showed significant (*p* < 0.05) changes during storage. For instance, where 1-pentanol, 1-hexanol, and 1-octen-3-ol were at a maximum at 5 °C/month 4, the maximum evolution of 1-penten-3-ol, *Z*-2-penten-1-ol, and propanol were recorded at 23 °C/month 4 and 23 °C/month 3, respectively, and they were 3.32, 1.60, and 5.30 times higher, compared to observations at 5 °C. Kernel volatile data obtained from Chandler showed no significant (*p* > 0.05) storage temperature effects, with respect to heptanal, *E*-2-hexenal, and octanal, but reflected an influence of storage time.

For all investigated walnut cultivars and storage conditions, this study showed hexanal and 1-hexanol as the highest elicited aldehyde and alcohol. This can be attributed to the significant distribution of oleic and linolenic acids among profiled fatty acids. These fatty acids are prone to oxidation, giving rise to aldehydes such as hexanal [40]. It is also interesting to highlight that, contrary to a recent work published by our research group on volatiles from Howard and Chandler kernels [13], the current study observed the presence of 1-octen-3-one in Howard (76.51–343.93 mg/kg) and Chandler (46.35–498.15 mg/kg) kernels, which was absent in our previous work. This metallic and mushroom aroma descriptor compound was observed in walnuts grown in China, Chile, and Ukraine [41], hence indicating the impact of cultivational and geographical conditions on the formation of volatile compounds.

### 3.5. Oil Volatile Changes

Twenty-three volatiles were detected in oils from both cultivars, although their concentrations were significantly lower (*p* > 0.05), compared to kernels. Of the 23 profiled volatiles, five (i.e., *E*-*E*-2,4-nonadienal, 1-penten-3-ol, ethyl acetate, propanoic acid, and hexanoic acid) were peculiar to kernels, and two (i.e., 3-carene and pentanoic acid) were peculiar to oil. From Table 6, the accumulation of hexanal, 1-octen-3-ol, and 1-hexanol were significantly (*p* < 0.05) higher at 5 °C/month 4 than with 23 °C/month 4 by 40.51, 45.38, and 87.81%, respectively, in Howard oil.

Volatiles from Chandler oil (Table 7) showed significant levels of 1-pentanol (52.96 mg/kg) and 1-octen-3-one (669.38 mg/kg) at 23 °C/month 3 and 5 °C/month 4, respectively. Additionally, the highest mean values for hexanal and 1-octen-3-ol in Chandler oil were recorded at 5 °C/month 4, but these values were statistically insignificant (*p* > 0.05), compared to 23 °C/month 4. Overall, the results showed volatile concentrations to be dependent on cultivar, walnut fraction, storage temperature, and time. Since these volatiles are associated with distinct aroma descriptors, it can be postulated that, storage conditions impact different odor perceptions in walnut oil and kernel. For instance, the presence of hexanal and 1-hexanol in oils signify tallow and resin odors during storage [42].

### 3.6. Impact of Storage on Sensory Attributes

**Figure 3:**
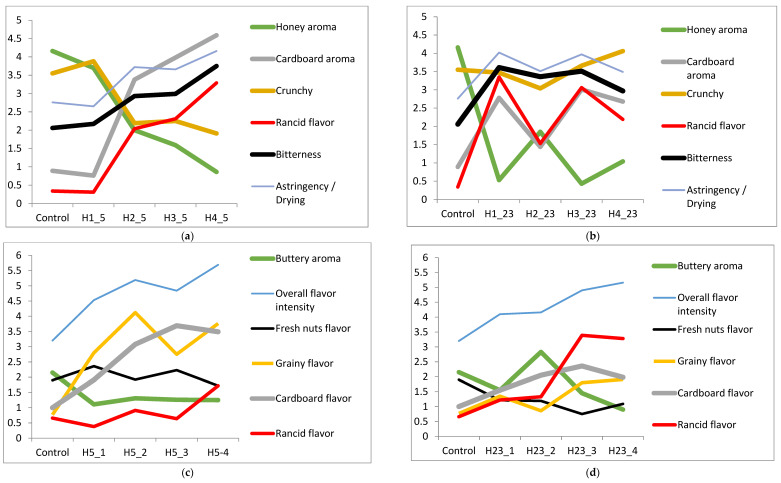
(**a**–**d**) Changes in sensory attributes of Howard kernel ((**a**) 5 °C; (**b**) 23 °C) and oil ((**c**) 5 °C; (**d**) 23 °C) for 4 months.

Changes in sensory properties of walnut kernels and oils are presented in Tables S1 and S2 of Supplementary Material. Compared to control kernels, Howard kernels stored at 5 °C/month 1 showed comparable scores for crunchiness, as well as honey and cardboard aromas. However, this observation decreased significantly (*p* < 0.05) from months 2 to 4 (Figure 3a–d). Contrary to kernels stored at 5 °C, the Howard kernels stored at 23 °C showed significant (*p* < 0.05) perceptions for rancid taste, rancid aroma, astringency, and bitterness as early as month 1. Linking this trend to its pre-discussed volatile data, it can be postulated that the rancidity at 5 °C is detected when the propanoic acid, octanal, *E*-2-heptenal, pentanal, and hexanal reach 0.50, 1.17, 2.18, 35.95, and 766.62 mg/kg, respectively, whereas rancidity at 23 °C is detected when the aforementioned volatiles reach levels of 0.29, 0.57, 1.65, 14.11, and 153.59 mg/kg, respectively.

This further indicates that hexanal is the dominating volatile for kernel rancidity detection, although it also exhibits synergistic actions with other rancid-inducing compounds. With respect to Howard oils, storage at 5 °C did not show rancidity until month 4, whereas oil at 23 °C was perceived rancid from month 3. Thus, the rancidity of Howard oil at 5 °C can be detected when oxidative volatiles, such as octanal, *E*-2-heptenal, butanal, pentanal, propanol, and hexanal reach 1.18, 2.94, 4.59, 25.22, 122.14, and 993.69 mg/kg, respectively, whereas, at 23 °C, oil rancidity is detected when the cited volatiles reach 0.68, 5.34, 3.73, 43.43, 90.48, and 675.32 mg/kg, respectively.

**Figure 4:**
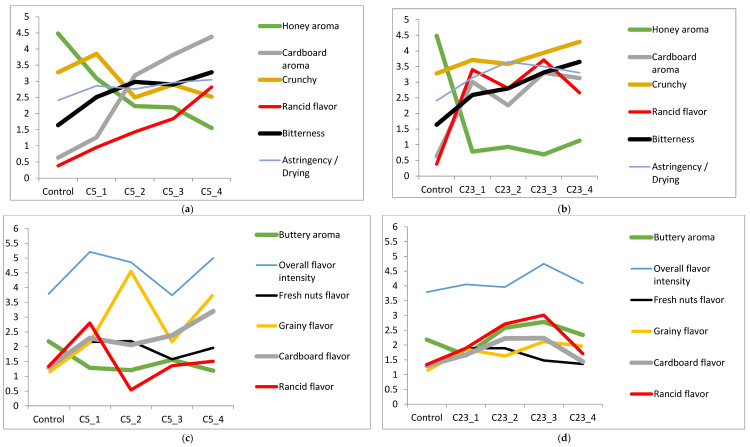
(**a**–**d**) Changes in sensory attributes of Chandler kernel ((**a**) 5 °C; (**b**) 23 °C) and oil ((**c**) 5 °C; (**d**) 23 °C) for 4 months.

For Chandler, kernels stored at 23 °C recorded the lowest scores for honey aroma, compared to kernels stored at 5 °C (Figure 4a–d). Additionally, bitterness and astringency in Chandler kernels were detected at early storage periods of 23 °C and late storage periods of 5 °C. Based on the sensory and volatile data, rancidity in Chandler kernels can be detected when concentrations of oxidative volatiles, such as *E-E*-2,4-nonadienal, propanoic acid, octanal, pentanal, *E*-2-heptenal, butanal, propanol, and hexanal reach 0.09, 0.41, 0.51, 1.53, 2.22, 2.25, 22.30, and 279.43 mg/kg, respectively. In addition, the detection of oil rancid flavor in Chandler at 23 °C/month 3 received the highest score. Thus, rancidity in Chandler oil was perceived at 23 °C, when octanal, butanal, *E*-2-heptenal, pentanal, propanol, and hexanal reached 1.00, 4.58, 6.28, 55.68, 93.23, and 954.13 mg/kg, respectively. Considering the increasing rate of hexanal with rancidity detection, hexanal is postulated to be the most appropriate volatile marker for walnut quality prediction. Mexis et al. [43] and Jensen et al. [12] also investigated storage impact on walnut quality. Their studies revealed positive correlations between the increasing hexanal production and sensory perception of bitterness and rancidity.

### 3.7. Correlations between Oxidative Volatiles and Sensory Attributes of Kernels and Oils

To further analyze the relationship between volatile compounds and sensory attributes, Pearson correlation coefficients were obtained with values of 22 volatiles (Table S3, Supplementary Material). For bitterness of kernels at 5 °C, five dominating volatiles that showed strong and positive correlations included hexanal (*r* = 0.918, *p <* 0.*01*), nonanal (*r* = 0.933, *p <* 0.*01*), 1-hexanol (*r* = 0.943, *p <* 0.*01*), *E*-2-pentenal (*r* = 0.939, *p <* 0.*01*), and 1-octen-3-ol (*r* = 0.950, *p <* 0.*01*). Astringency at 5 °C showed high positive correlations, with 19 volatile compounds, of which, the four most highly correlated were 1-hexanol (*r* = 0.921, *p <* 0.*01*), nonanal (*r* = 0.865, *p <* 0.*01*), 1-pentanol (*r* = 0.877, *p <* 0.*01*), and 1-octen-3-ol (*r* = 0.866, *p <* 0.*01*). Additionally, rancid aroma was positively correlated with 19 volatiles, with the highest correlating compounds being nonanal (*r* = 0.903, *p <* 0.*01*), hexanal (*r* = 0.843, *p <* 0.*01*), 1-hexanol (*r* = 0.968, *p <* 0.*01*), 1-pentanol (*r* = 0.933, *p <* 0.*01*), and pentanal (*r* = 0.872, *p <* 0.*01*). This study confirmed a direct relationship between the increasing oxidative volatiles and reduction of walnut kernel oxidative stability.

Unlike that observed with kernels, the oxidative stability of walnut oils showed higher positive correlations with volatiles at 23 °C than at 5 °C (Table S4, Supplementary Material). At 5 °C, rancid flavor and rancid aroma of oils were strongly correlated with only hexanal (*r* = 0.922, *p <* 0.*01*; *r* = 0.920, *p <* 0.*01*, respectively). However, at 23 °C, rancid flavor and aroma were positively correlated with 17 and 19 volatiles, respectively. Pentanal showed the highest positive correlation (*r* = 0.881, *p <* 0.*01*) with rancid oil flavor at 23 °C, whereas 5-hepten-2-one and 6-methyl were the most highly positively correlated (*r* = 0.918, *p <* 0.*01*) with rancid aroma, followed by hexanal (*r* = 0.845, *p <* 0.*01*) and pentanal (*r* = 0.849, *p <* 0.*01*). In summary, rancidity in walnut oils at 5 °C was highly linked with hexanal, whereas rancidity at 23 °C was not only linked with hexanal, but also the synergistic activities of other oxidative volatiles.

### 3.8. Partial Least Squares (PLS) Regression Analysis

**Figure 5:**
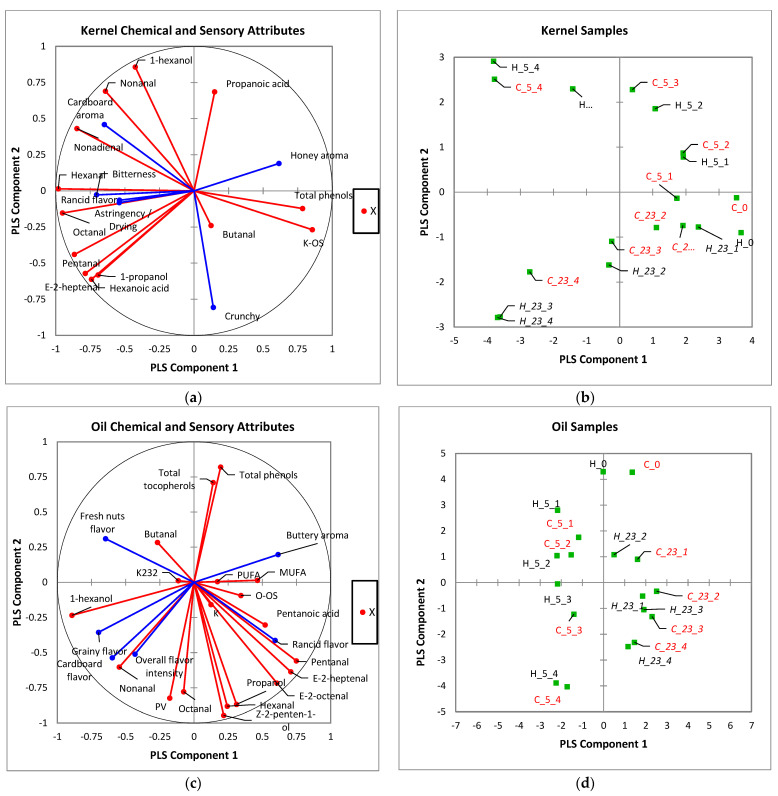
(**a**–**d**) Graphic explanation of the relationship between chemical oxidative markers and development of rancidity in walnut kernel (**a**,**b**) and oil (**c**,**d**) during storage by partial least squares regression analysis.

PLS regression analysis was employed to evaluate the relationship between sensory perception and oxidative chemical markers. Figure 5a display the results for the chemical oxidative markers (*x*-variable) and sensory attributes (*y*-variable) of kernels and their loading plots (Figure 5b).

As presented in the positive quadrant of PLS component 1 of Figure 5a, kernel total phenolic content, butanal, and oxidative stability correlated positively with the sensory perceptions of honey aroma and crunchiness. This explains the close clustering of all control and kernels stored at early periods (i.e., months 1 and 2) in Figure 5b. Additionally, there was a clear progression of 5 °C stored kernels, from a honey aroma to cardboard aroma, whereas 23 °C stored kernels drifted from honey aroma to crunchy and then to rancid, bitter, and astringent. Thus, it was not surprising that sensory perception of cardboard aroma showed a close relationship with nonanal, nonadienal, and 1-hexanol, whereas rancidity, bitterness, and astringency showed a positive correlation with hexanal, octanal, and pentanal.

For oil, PLS regression analysis indicates that samples stored at 23 °C were clustered in the positive quadrant (Figure 5d), with a loss of buttery aroma into the progression of rancidity, as the key sensory attribute. This trend correlates positively with the distribution of hexanal and pentanal (Figure 5c). Nevertheless, oils stored at 5 °C did not depict sensory rancidity but showed a progression from fresh nut flavor to cardboard and grainy flavors, which resulted in a low overall flavor intensity. Additionally, the fresh nut flavor of oils stored at early periods of 5 °C showed a close relationship with butanal, whereas the perceptions of cardboard and grainy flavors correlated with 1-hexanol and nonanal. It was also interesting to observe that all control samples showed strong and positive relationships with antioxidant compounds and stability indices, irrespective of the walnut fraction.

## 4. Conclusions

In summary, the popularity of walnuts has increased as consumers recognize their health benefits as nutrient-dense foods. However, the poor management of post-harvest practices, such as storage temperature and time, can lead to losses in walnut nutritional quality and organoleptic characteristics. This study showed that the sensory attributes and health-promoting biomarkers can be retained for up to three months when walnuts are stored at 5 °C. Sensory profiling also showed that the perception of rancidity in walnut oils is dependent on the storage time and temperature. Furthermore, Pearson’s correlation test showed positive correlations between oxidative volatiles and the stability of walnut kernels and oils, where kernel rancid perception was strongly linked with volatiles at 5 °C (i.e., due to high moisture content), and oil rancid perception was strongly linked with volatiles produced at 23 °C. Additionally, the study revealed high positive correlations between bitterness, astringency, rancidity, and oxidative volatiles, such as hexanal, pentanal, and propanol. To maintain the positive sensory attributes and market value of walnuts, while extending their shelf-life, it is recommended to store oxidation-prone cultivars, such as Howard, at lower temperatures, compared to oxidation-resistant cultivars, such as Chandler.

## Figures and Tables

**Table 1:** Changes in quality parameters during storage.

Cultivar	Temp. (C)	Storage Time (Months)	Quality Parameter						
			Moisture (g/kg)	FFA (g/100 g)	Oil PV (meq O_2_/kg)	K_232_	K_268_	Kernel OS (h)	Oil OS (h)
Howard									
		0	4.13 ± 0.3d	0.01 ± 0.0c	0.85 ± 0.1c	0.90 ± 0.0e	0.09 ± 0.0f	14.08 ± 0.11a	2.84 ± 0.07a
	5 °C	1	4.40 ± 0.2d	0.02 ± 0.0b	0.98 ± 0.2bc	1.25 ± 0.0cb	0.13 ± 0.0c	13.10 ± 0.34a	2.84 ± 0.05a
		2	6.87 ± 0.6b	0.02 ± 0.0b	1.06 ± 0.2b	1.28 ± 0.0cb	0.15 ± 0.0b	12.07 ± 0.19bc	2.68 ± 0.03b
		3	7.57 ± 0.3a	0.03 ± 0.0a	1.23 ± 0.1b	1.35 ± 0.0b	0.15 ± 0.0b	11.44 ± 0.52c	2.46 ± 0.01cd
		4	7.88 ± 0.2a	0.03 ± 0.0a	1.80 ± 0.0a	1.55 ± 0.0b	0.10 ± 0.0e	10.74 ± 0.37d	2.51 ± 0.01c
	23 °C	1	5.4 ± 0.2c	0.02 ± 0.0b	0.93 ± 0.3bc	1.42 ± 0.0d	0.12 ± 0.0d	14.07 ± 0.04a	2.88 ± 0.02a
		2	4.4 ± 0.0d	0.02 ± 0.0b	1.13 ± 0.1b	1.56 ± 0.0c	0.13 ± 0.0cd	12.59 ± 0.24b	2.65 ± 0.02b
		3	5.07 ± 0.2c	0.02 ± 0.0b	1.06 ± 0.1b	1.82 ± 0.2b	0.18 ± 0.0a	12.22 ± 0.33bc	2.57 ± 0.02c
		4	5.16 ± 0.6c	0.03 ± 0.0a	1.80 ± 0.2a	2.50 ± 0.1a	0.18 ± 0.0a	11.07 ± 0.50cd	2.48 ± 0.01d
Chandler									
		0	4.22 ± 0.3d	0.01 ± 0.0c	0.70 ± 0.2d	1.06 ± 0.0g	0.09 ± 0.0b	14.54 ± 0.50a	2.97 ± 0.13a
	5 °C	1	4.33 ± 0.3d	0.02 ± 0.0b	1.06 ± 0.1c	1.24 ± 0.0edf	0.14 ± 0.0a	13.97 ± 0.17ba	2.87 ± 0.02a
		2	6.93 ± 0.5a	0.02 ± 0.0b	1.06 ± 0.2c	1.21 ± 0.0e	0.10 ± 0.0b	13.09 ± 0.10c	2.68 ± 0.02b
		3	6.20 ± 0.1b	0.02 ± 0.0b	1.33 ± 0.1bc	1.29 ± 0.0c	0.10 ± 0.0b	12.33 ± 0.49d	2.39 ± 0.05c
		4	7.42 ± 0.1a	0.03 ± 0.0a	1.50 ± 0.1ab	1.39 ± 0.0bc	0.09 ± 0.0b	12.07 ± 0.53d	2.28 ± 0.06d
	23 °C	1	5.27 ± 0.6bc	0.01 ± 0.0c	0.93 ± 0.3bd	1.27 ± 0.0e	0.13 ± 0.0a	14.01 ± 0.42a	2.72 ± 0.04a
		2	4.73 ± 0.6cd	0.02 ± 0.0cb	1.13 ± 0.1b	1.45 ± 0.1d	0.11 ± 0.0b	13.06 ± 0.05b	2.66 ± 0.05a
		3	5.26 ± 0.3bc	0.02 ± 0.0b	1.06 ± 0.1b	1.68 ± 0.1b	0.11 ± 0.0b	13.07 ± 0.13b	2.51 ± 0.03b
		4	4.37 ± 0.3d	0.03 ± 0.0a	1.80 ± 0.2a	1.94 ± 0.0a	0.13 ± 0.0a	12.24 ± 0.24c	2.03 ± 0.03c

Data are presented as mean ± SD of three independent experiments. Different letters within a column indicate significant differences (*p* < 0.05) for each cultivar.

**Table 2:** Changes in phenolic composition of walnut kernels during storage.

Cultivar	Phenolic Compound (mg/kg)	5 °C					23 °C			
Howard		Month 0	Month 1	Month 2	Month 3	Month 4	Month 1	Month 2	Month 3	Month 4
	Total phenols	11,119.13 ± 585.3a	11,508.64 ± 342.8a	10,852.22 ± 131.5bc	9645.08 ± 114.0bc	9094.22 ± 185.7bc	12,092.99 ± 131.9a	10,386.31 ± 102.2bc	9997.83 ± 105.9bc	10,102.79 ± 519.3c
	Gallic acid	467.47 ± 24.9e	363.11 ± 0.9d	616.18 ± 1.2b	516.31 ± 0.6c	513.8 ± 32.3ce	651.47 ± 0.5b	568.10 ± 1.2c	549.49 ± 1.7c	703.03 ± 17.1a
	Gallocatechin	20.87 ± 7.8g	29.66 ± 0.9f	77.50 ± 0.9a	61.28 ± 0.6b	41.84 ± 0.9d	76.18 ± 0.4a	57.50 ± 1.0c	62.03 ± 1.7b	34.50 ± 2.9e
	Protocatechuic acid	54.74 ± 9.4b	68.29 ± 1.0a	40.02 ± 1.2c	35.45 ± 0.6d	27.60 ± 0.7e	40.60 ± 0.5c	51.94 ± 0.8b	29.52 ± 1.8e	20.74 ± 0.5f
	Catechin	13.91 ± 2.9e	37.30 ± 1.4b	23.39 ± 0.9c	21.50 ± 0.6d	18.25 ± 1.2e	25.40 ± 0.9c	51.46 ± 0.8a	25.06 ± 1.7c	35.68 ± 1.9b
	Chlorogenic acid	43.59 ± 3.9b	50.66 ± 1.0a	33.06 ± 1.2c	20.98 ± 0.6e	42.36 ± 16.6ab	31.69 ± 0.7c	27.06 ± 0.9d	23.82 ± 1.5d	54.38 ± 1.1a
	Vanillic acid	56.35 ± 1.9f	197.05 ± 0.8a	86.22 ± 1.2b	57.94 ± 0.6f	60.02 ± 1.7e	87.58 ± 0.6b	75.34 ± 1.3d	62.70 ± 1.8e	81.53 ± 1.1c
	Caffeic acid	43.40 ± 8.9i	125.03 ± 0.9h	222.16 ± 1.2b	182.79 ± 0.6f	207.58 ± 7.2d	211.61 ± 0.6c	190.92 ± 1.1e	166.35 ± 1.6g	240.62 ± 9.1a
	Epicatechin	31.72 ± 6.2e	67.41 ± 0.7a	50.51 ± 0.8bc	44.41 ± 0.6d	40.77 ± 6.6d	51.73 ± 0.8bc	48.14 ± 0.8c	53.90 ± 1.8b	32.84 ± 2.9e
	Gallocatechin gallate	10.78 ± 0.2h	32.29 ± 0.9g	79.44 ± 1.5e	62.60 ± 0.6f	185.99 ± 42.1a	81.84 ± 0.5de	86.79 ± 0.8d	129.42 ± 2.1c	173.06 ± 0.8b
	*p*-coumaric acid	134.85 ± 0.4c	137.76 ± 1.2c	161.18 ± 1.2a	124.17 ± 0.6d	49.07 ± 44.7f	148.77 ± 0.6b	121.11 ± 1.0d	106.21 ± 2.0e	31.05 ± 2.7g
	Epicatechin gallate	489.97 ± 78.9b	667.30 ± 0.9a	194.54 ± 0.9ce	182.15 ± 0.6d	164.17 ± 25.9e	187.43 ± 0.5de	181.38 ± 1.1d	189.16 ± 2.1de	86.15 ± 11.8f
	Rutin	139.29 ± 18.9h	226.92 ± 1.1g	671.04 ± 1.2b	559.58 ± 0.6e	53.03 ± 0.6i	680.72 ± 0.5a	635.77 ± 1.2c	604.13 ± 1.5d	256.56 ± 4.7f
	Quercetin	203.16 ± 1.7b	165.57 ± 0.9e	194.69 ± 1.1c	132.64 ± 0.6f	160.53 ± 9.8e	191.57 ± 0.2c	183.19 ± 0.8d	196.28 ± 1.9bc	247.73 ± 2.5a
	Ellagic acid	45.98 ± 4.3f	49.20 ± 1.2f	96.11 ± 1.2c	69.74 ± 0.6e	141.42 ± 7.4a	85.61 ± 0.9d	123.69 ± 1.3b	88.62 ± 2.1c	154.86 ± 2.8a
**Chandler**										
	Total Phenol	12,423.75 ± 401.4a	11,757.97 ± 224.6a	11,553.24 ± 293.1a	9855.02 ± 91.3b	8939.90 ± 202.6d	11,449.12 ± 298.9a	9464.39 ± 147.1c	8988.20 ± 95.5d	9077.50 ± 365.8d
	Gallic acid	243.37 ± 20.4h	506.34 ± 0.8f	775.09 ± 1.4b	599.15 ± 0.6d	639.52 ± 19.7c	382.27 ± 0.9g	612.84 ± 1.3c	545.02 ± 1.3e	981.68 ± 84.3a
	Gallocatechin	27.01 ± 2.5f	15.04 ± 0.8g	93.99 ± 1.9a	51.44 ± 0.6c	27.89 ± 2.4e	21.45 ± 1.1f	72.52 ± 1.1b	47.31 ± 1.3d	31.44 ± 0.6e
	Protocatechuic acid	73.57 ± 5.0a	76.96 ± 0.9a	35.26 ± 1.1b	25.89 ± 0.6c	27.89 ± 2.4c	80.38 ± 1.1a	35.25 ± 1.1b	26.76 ± 1.3c	17.59 ± 1.2d
	Catechin	40.01 ± 3.2c	34.15 ± 1.1d	54.11 ± 1.3b	57.02 ± 0.6b	35.45 ± 4.1d	20.18 ± 1.1e	63.89 ± 0.9a	20.91 ± 1.2e	52.86 ± 18.4b
	Chlorogenic acid	439.40 ± 39.4a	42.68 ± 0.8b	24.22 ± 1.7de	22.60 ± 1.2de	17.34 ± 0.8e	32.60 ± 1.2c	27.52 ± 1.2dc	24.07 ± 1.0dec	20.26 ± 0.8e
	Vanillic acid	68.02 ± 3.3ef	89.42 ± 0.7bc	94.95 ± 1.2ab	85.51 ± 0.6cd	74.25 ± 2.3e	73.40 ± 1.1e	80.28 ± 0.7e	60.75 ± 0.9f	121.88 ± 11.5a
	Caffeic acid	250.36 ± 59.9cd	234.49 ± 0.8e	316.65 ± 1.4bc	250.67 ± 0.6de	279.03 ± 3.5d	124.90 ± 1.1f	325.44 ± 1.0bc	228.99 ± 1.4e	498.03 ± 32.9a
	Epicatechin	57.67 ± 18.4c	62.19 ± 0.6a	39.77 ± 1.7bc	48.32 ± 0.6b	33.53 ± 2.7c	35.04 ± 0.9c	39.40 ± 1.2c	40.35 ± 1.3c	33.95 ± 1.1c
	Gallocatechin gallate	34.09 ± 0.3f	12.32 ± 0.8g	76.76 ± 1.5c	60.42 ± 0.6d	124.43 ± 19.6b	50.62 ± 1.2e	49.43 ± 0.9e	57.58 ± 0.9de	208.41 ± 9.8a
	*p*-coumaric acid	190.62 ± 25.8abc	134.70 ± 0.9bf	159.01 ± 1.1c	142.08 ± 0.6e	166.91 ± 9.5b	153.77 ± 1.1dc	132.87 ± 0.8f	111.47 ± 1.2g	244.77 ± 17.4a
	Epicatechin gallate	683.94 ± 59.6a	620.58 ± 0.8a	187.68 ± 1.8c	171.13 ± 0.6d	185.96 ± 1.4c	413.22 ± 1.2b	175.80 ± 1.1d	186.96 ± 1.3c	183.27 ± 5.6c
	Rutin	513.06 ± 42.3d	134.81 ± 0.9e	708.09 ± 1.8b	541.23 ± 0.6d	569.99 ± 23.0d	212.56 ± 1.1f	630.56 ± 1.2c	509.11 ± 0.9d	891.73 ± 56.8a
	Quercetin	203.69 ± 24.9dc	215.44 ± 0.8d	175.92 ± 1.6de	267.91 ± 0.6b	167.87 ± 6.3e	300.27 ± 0.9a	156.42 ± 0.9f	143.05 ± 0.8f	259.21 ± 17.0c
	Ellagic acid	53.57 ± 5.7cd	50.17 ± 0.9d	59.88 ± 1.5bc	54.38 ± 0.6d	47.86 ± 3.4e	49.87 ± 1.3e	76.34 ± 1.1a	45.40 ± 1.5ef	44.86 ± 0.9f

Data are presented as mean ± SD of three independent experiments. Different letters within a row indicate significant differences (*p* < 0.05) for each cultivar.

**Table 3:** Changes in oil fatty acids profile during storage.

Cultivar	Fatty Acid (%)	5 °C					23 °C			
Howard		Month 0	Month 1	Month 2	Month 3	Month 4	Month 1	Month 2	Month 3	Month 4
	C16:0	2.29 ± 0.2a	2.88 ± 0.2a	2.38 ± 0.3a	2.29 ± 0.0a	2.28 ± 0.0a	1.75 ± 0.2a	2.61 ± 0.3a	1.66 ± 0.1a	1.69 ± 0.0a
	C16:1	6.43 ± 0.0a	6.24 ± 0.0a	6.28 ± 0.0a	6.33 ± 0.0a	6.32 ± 0.0a	6.29 ± 0.0a	6.29 ± 0.0a	6.37 ± 0.0a	6.37 ± 0.0a
	C17:0	0.06 ± 0.0a	0.06 ± 0.0a	0.06 ± 0.0a	0.06 ± 0.0a	0.06 ± 0.0a	0.06 ± 0.0a	0.06 ± 0.0a	0.06 ± 0.0a	0.06 ± 0.0a
	C17:1	2.32 ± 0.0a	2.20 ± 0.0a	2.39 ± 0.1a	2.34 ± 0.0a	2.34 ± 0.0a	2.29 ± 0.1a	2.20 ± 0.1a	2.35 ± 0.3a	2.34 ± 0.0a
	C18:0	3.69 ± 0.1a	4.72 ± 0.3a	4.43 ± 0.9a	4.11 ± 0.0a	4.10 ± 0.0a	4.11 ± 0.2a	4.33 ± 0.1a	3.62 ± 0.3a	3.64 ± 0.3a
	C18:1	9.20 ± 0.2a	8.42 ± 0.3a	8.57 ± 0.9a	8.92 ± 0.2a	8.93 ± 0.2a	8.99 ± 0.3a	8.56 ± 0.3a	9.49 ± 0.3a	9.33 ± 0.1a
	C18:2	59.83 ± 0.1a	59.61 ± 0.2a	59.88 ± 0.3a	60.08 ± 0.1a	60.11 ± 0.1a	60.46 ± 0.1a	59.99 ± 0.1a	60.58 ± 0.0a	60.69 ± 0.1a
	C18:3	15.87 ± 0.0a	15.56 ± 0.0a	15.71 ± 0.1a	15.56 ± 0.0a	15.56 ± 0.0a	15.74 ± 0.0a	15.63 ± 0.0a	15.54 ± 0.0a	15.56 ± 0.1a
	C22:0	0.09 ± 0.0a	0.09 ± 0.0a	0.09 ± 0.0a	0.09 ± 0.5a	0.08 ± 0.0a	0.08 ± 0.0a	0.08 ± 0.0a	0.09 ± 0.0a	0.09 ± 0.0a
	C22:1	0.23 ± 0.0a	0.23 ± 0.0a	0.23 ± 0.0a	0.23 ± 0.0a	0.23 ± 0.0a	0.23 ± 0.0a	0.23 ± 0.0a	0.23 ± 0.0a	0.22 ± 0.0a
	SFA	6.13 ± 0.3a	7.75 ± 0.5a	6.95 ± 1.2a	6.54 ± 0.0a	6.51 ± 0.0a	5.99 ± 0.4a	7.08 ± 0.4a	5.43 ± 0.2a	5.48 ± 0.2a
	UFA	93.87 ± 0.3a	92.25 ± 0.5a	93.05 ± 1.2a	93.46 ± 0.0a	93.49 ± 0.0a	94.01 ± 0.4a	92.92 ± 0.4a	94.57 ± 0.2a	94.52 ± 0.2a
	MUFA	18.18 ± 0.2a	17.08 ± 0.3a	17.46 ± 0.9a	17.82 ± 0.2a	17.81 ± 0.2a	17.81 ± 0.2a	17.29 ± 0.2a	18.45 ± 0.3a	18.27 ± 0.1a
	PUFA	75.70 ± 0.1a	75.17 ± 0.2a	75.59 ± 0.4a	75.64 ± 0.1a	75.67 ± 0.1a	76.19 ± 0.2a	75.63 ± 0.2a	76.12 ± 0.0a	76.25 ± 0.2a
**Chandler**										
	C16:0	2.18 ± 0.5a	1.59 ± 0.5a	1.80 ± 0.0a	2.48 ± 0.2a	2.45 ± 0.1a	2.35 ± 0.1a	1.97 ± 0.0a	2.42 ± 0.2a	2.42 ± 0.2a
	C16:1	5.89 ± 0.0a	5.93 ± 0.0a	6.00 ± 0.0a	5.98 ± 0.0a	6.03 ± 0.1a	6.02 ± 0.0a	5.84 ± 0.2a	6.07 ± 0.0a	6.07 ± 0.0a
	C17:0	0.06 ± 0.0a	0.06 ± 0.0a	0.06 ± 0.0a	0.06 ± 0.0a	0.06 ± 0.0a	0.06 ± 0.0a	0.04 ± 0.0a	0.06 ± 0.0a	0.06 ± 0.0a
	C17:1	1.32 ± 1.7a	2.42 ± 0.1a	2.18 ± 0.1a	2.42 ± 0.1a	2.32 ± 0.0a	2.41 ± 0.1a	2.46 ± 0.0a	2.33 ± 0.0a	2.33 ± 0.0a
	C18:0	3.26 ± 1.3a	4.53 ± 0.5a	4.42 ± 0.0a	3.96 ± 0.2a	4.28 ± 0.3a	3.86 ± 0.1a	4.05 ± 0.2a	4.24 ± 0.3a	4.24 ± 0.3a
	C18:1	11.77 ± 2.9a	9.63 ± 0.5a	9.58 ± 0.1a	9.97 ± 0.1a	9.58 ± 0.4a	9.77 ± 0.1a	9.88 ± 0.2a	9.52 ± 0.4a	9.52 ± 0.4a
	C18:2	60.38 ± 0.3a	60.65 ± 0.2a	60.81 ± 0.1a	60.29 ± 0.2a	60.35 ± 0.1a	60.25 ± 0.0a	60.74 ± 0.1a	60.36 ± 0.1a	60.36 ± 0.1a
	C18:3	14.84 ± 0.1a	14.88 ± 0.1a	14.82 ± 0.0a	14.52 ± 0.1a	14.61 ± 0.1a	14.97 ± 0.0a	14.69 ± 0.0a	14.69 ± 0.0a	14.69 ± 0.0a
	C22:0	0.08 ± 0.0a	0.09 ± 0.0a	0.09 ± 0.0a	0.09 ± 0.0a	0.09 ± 0.0a	0.08 ± 0.0a	0.08 ± 0.0a	0.09 ± 0.0a	0.09 ± 0.0a
	C22:1	0.23 ± 0.0a	0.24 ± 0.0a	0.24 ± 0.0a	0.24 ± 0.0a	0.24 ± 0.0a	0.23 ± 0.0a	0.24 ± 0.0a	0.24 ± 0.0a	0.24 ± 0.0a
	SFA	5.57 ± 0.8a	6.26 ± 0.1a	6.37 ± 0.0a	6.59 ± 0.0a	6.87 ± 0.4a	6.35 ± 0.0a	6.14 ± 0.2a	6.80 ± 0.5a	6.80 ± 0.5a
	UFA	94.43 ± 0.8a	93.74 ± 0.1a	93.63 ± 0.0a	93.41 ± 0.0a	93.13 ± 0.4a	93.65 ± 0.0a	93.86 ± 0.2a	93.20 ± 0.5a	93.20 ± 0.5a
	MUFA	19.20 ± 1.2a	18.21 ± 0.3a	17.99 ± 0.1a	18.60 ± 0.2a	18.18 ± 0.1a	18.43 ± 0.0a	18.42 ± 0.1a	18.16 ± 0.4a	18.16 ± 0.4a
	PUFA	75.23 ± 0.4a	75.53 ± 0.3a	75.63 ± 0.1a	74.81 ± 0.2a	74.95 ± 0.1a	75.22 ± 0.0a	75.44 ± 0.1a	75.04 ± 0.1a	75.04 ± 0.1a

Data are presented as mean ± SD of three independent experiments. Different letters within a row indicate significant differences (*p* < 0.05) for each cultivar.

**Table 4:** Volatile changes in Howard kernels during storage.

Volatile Compound (mg/kg)	5 °C					23 °C			
Month	0	1	2	3	4	1	2	3	4
Propanol	2.17 ± 0.6h	8.42 ± 0.1g	6.99 ± 0.4f	18.16 ± 1.9e	31.99 ± 6.3d	13.87 ± 1.7e	59.00 ± 7.1c	90.82 ± 12.7b	169.77 ± 30.6a
Butanal	28.55 ± 1.9a	2.44 ± 0.5fe	2.39 ± 0.3fe	3.42 ± 0.1e	4.36 ± 0.7de	1.42 ± 0.3g	5.08 ± 0.6d	6.99 ± 0.4c	9.18 ± 1.4b
Pentanal	1.33 ± 0.1g	11.86 ± 0.3f	12.47 ± 1.3f	35.95 ± 2.2e	48.02 ± 6.2d	14.11 ± 1.3f	64.21 ± 5.3c	109.22 ± 5.5a	88.82 ± 13.8b
Hexanal	22.77 ± 2.1f	205.66 ± 31.9d	260.37 ± 70.7d	766.62 ± 33.3b	1319.45 ± 54.8a	153.59 ± 18.5e	585.90 ± 52.7c	1227.73 ± 39.6a	1238.37 ± 165.7a
*E*-2-Pentenal	0.11 ± 0.1g	0.50 ± 0.1dfg	0.66 ± 0.0efg	1.11 ± 0.1ef	1.70 ± 0.2d	1.10 ± 0.2ef	3.70 ± 0.3c	6.58 ± 0.3b	10.25 ± 0.9a
Heptanal	0.32 ± 0.1e	1.65 ± 0.2cd	2.11 ± 0.1c	4.98 ± 0.6b	9.32 ± 1.3a	1.12 ± 0.1de	3.56 ± 0.2b	7.88 ± 0.1a	7.09 ± 0.9a
*E*-2-Hexenal	0.21 ± 0.0e	1.02 ± 0.1d	1.61 ± 0.1d	5.16 ± 0.2b	7.50 ± 0.9a	1.50 ± 0.1d	3.65 ± 0.3c	7.06 ± 0.5a	4.93 ± 0.6b
Octanal	0.19 ± 0.0c	0.72 ± 0.1b	0.62 ± 0.1b	1.17 ± 0.0b	1.97 ± 0.2a	0.57 ± 0.1b	1.05 ± 0.1b	2.53 ± 0.0a	2.45 ± 0.9a
*E*-2-Heptenal	0.30 ± 0.0f	0.75 ± 0.0e	0.77 ± 0.1e	2.18 ± 0.1d	13.20 ± 0.6a	1.65 ± 0.1d	6.84 ± 0.2b	13.20 ± 0.6a	4.38 ± 0.9c
Nonanal	0.52 ± 0.0d	1.76 ± 0.1d	2.81 ± 0.2c	5.74 ± 0.9b	7.98 ± 0.6a	0.95 ± 0.1d	1.03 ± 0.1d	2.27 ± 0.5c	2.89 ± 0.2c
*E*-2-Octenal	0.21 ± 0.0f	0.53 ± 0.1e	0.70 ± 0.1e	2.64 ± 0.2d	7.63 ± 1.6b	1.15 ± 0.1e	4.16 ± 0.2c	11.52 ± 0.3a	6.27 ± 0.9b
Benzaldehyde	1.23 ± 0.0d	1.32 ± 0.1cd	1.87 ± 0.1a	1.68 ± 0.1b	1.43 ± 0.1c	1.15 ± 0.1d	1.57 ± 0.0c	1.80 ± 0.1a	1.87 ± 0.2a
*E-E*-2,4-Nonadienal	0.02 ± 0.0e	0.21 ± 0.0d	0.26 ± 0.0d	0.73 ± 0.0cb	1.59 ± 0.2a	0.09 ± 0.0e	0.25 ± 0.0d	0.81 ± 0.0b	0.58 ± 0.0c
1-penten-3-ol	3.34 ± 0.1f	11.85 ± 0.6e	10.68 ± 0.4e	26.43 ± 1.7d	38.42 ± 2.5c	25.44 ± 1.1d	86.13 ± 4.4b	125.67 ± 11.1a	127.47 ± 17.4a
1-pentanol	1.31 ± 0.1f	18.12 ± 0.9e	38.74 ± 2.5d	89.79 ± 3.9b	115.59 ± 10.8a	14.32 ± 1.0e	43.64 ± 2.1d	89.43 ± 5.4b	62.54 ± 6.5c
*Z*-2-Penten-1-ol	0.11 ± 0.0e	0.41 ± 0.0d	0.60 ± 0.0d	1.06 ± 0.1c	2.51 ± 0.3b	0.47 ± 0.1d	1.11 ± 0.0c	2.27 ± 0.1b	4.01 ± 0.3a
1-hexanol	3.21 ± 0.3h	100.88 ± 4.9d	306.10 ± 22.7c	520.38 ± 33.6b	607.47 ± 66.7a	8.48 ± 0.3g	13.50 ± 0.5f	24.92 ± 1.4e	19.43 ± 3.7ef
1-octen-3-ol	0.51 ± 0.1i	7.07 ± 0.4g	11.02 ± 0.2f	18.69 ± 1.4c	33.01 ± 3.7a	3.19 ± 0.1h	12.52 ± 0.7e	26.73 ± 0.9b	15.79 ± 2.1d
Ethyl acetate	493.14 ± 32.2b	835.94 ± 33.7a	404.04 ± 32.2b	343.52 ± 19.9c	105.26 ± 6.1e	884.45 ± 26.7a	436.91 ± 9.5b	313.14 ± 43.5c	242.76 ± 37.3d
2-pentylfuran	1.37 ± 0.2f	4.95 ± 0.3e	8.58 ± 0.3d	17.75 ± 1.8c	24.39 ± 5.9a	3.62 ± 0.3e	8.51 ± 0.3c	17.76 ± 0.3b	14.14 ± 1.9b
1-octen-3-one	76.51 ± 10.5c	184.13 ± 44.8b	193.92 ± 85.4b	197.58 ± 42.2b	111.75 ± 38.5b	142.87 ± 74.7b	149.40 ± 47.2b	116.84 ± 16.2b	343.93 ± 62.7a
2-heptanone	0.40 ± 0.1e	1.37 ± 0.0d	1.85 ± 0.1d	5.96 ± 0.7b	8.71 ± 0.3a	1.41 ± 0.1d	4.50 ± 0.2c	8.34 ± 0.1a	5.95 ± 0.7b
6-methyl-5-hepten-2-one	0.31 ± 0.1de	1.17 ± 0.1b	1.86 ± 0.1ab	1.98 ± 0.3a	1.64 ± 0.2b	0.46 ± 0.0d	0.91 ± 0.0c	1.12 ± 0.1bc	1.08 ± 0.2bc
Propanoic acid	0.26 ± 0.1d	0.75 ± 0.2ab	0.82 ± 0.4ab	0.50 ± 0.2bc	0.45 ± 0.1c	0.29 ± 0.1d	0.22 ± 0.1d	0.25 ± 0.0d	0.26 ± 0.1d
Hexanoic acid	0.55 ± 0.1d	4.03 ± 0.8c	4.61 ± 0.5c	16.47 ± 1.9c	30.38 ± 8.1b	10.63 ± 1.7c	31.80 ± 4.5b	133.84 ± 5.6a	132.78 ± 12.9a
**Sum**	**638.96 ± 29.3c**	**1407.49 ± 65.6b**	**1276.46 ± 185.5b**	**2089.68 ± 44.2a**	**2526.88 ± 93.5a**	**1287.9 ± 107.9b**	**1529.15 ± 49.1b**	**2348.74 ± 108.3a**	**2522.4 ± 322.4a**

Data are presented as mean ± SD of three independent experiments. Different letters within a row indicate significant differences (*p* < 0.05) for each cultivar.

**Table 5:** Volatile changes in Chandler kernels during storage.

Volatile Compound (mg/kg)	5 °C					23 °C			
Month	0	1	2	3	4	1	2	3	4
Propanol	1.71 ± 0.2e	10.85 ± 1.6d	7.99 ± 0.4d	12.74 ± 1.8d	42.63 ± 5.6b	22.30 ± 1.7c	14.21 ± 0.3d	79.83 ± 8.9a	65.56 ± 16.5a
Butanal	5.83 ± 7.9abc	2.63 ± 0.7c	2.88 ± 1.0c	2.68 ± 0.4c	6.09 ± 0.8a	2.25 ± 0.2c	1.30 ± 0.1c	3.35 ± 1.10c	4.84 ± 0.8b
Pentanal	1.34 ± 0.2d	22.14 ± 3.2b	13.29 ± 0.4c	13.69 ± 1.1c	62.06 ± 9.6a	28.91 ± 3.9b	16.74 ± 1.2c	31.44 ± 5.2b	85.88 ± 15.6a
Hexanal	14.72 ± 1.1f	365.53 ± 53.8dc	231.83 ± 6.2d	423.67 ± 20.7c	1454.16 ± 208.3a	279.43 ± 41.1d	171.20 ± 7.9e	552.66 ± 26.3b	1079.22 ± 196.4b
*E*-2-Pentenal	0.09 ± 0.0d	0.53 ± 0.1c	0.55 ± 0.1c	0.69 ± 0.1c	1.81 ± 0.2b	1.53 ± 0.3b	1.79 ± 0.1b	4.81 ± 1.1a	5.02 ± 1.1a
Heptanal	0.44 ± 0.1e	1.66 ± 0.2c	2.24 ± 0.1c	4.05 ± 0.1b	9.36 ± 1.2a	1.67 ± 0.2c	1.06 ± 0.1d	2.94 ± 0.5c	6.17 ± 1.8a
*E*-2-Hexenal	0.35 ± 0.3d	1.16 ± 0.4c	1.39 ± 0.1c	3.99 ± 0.2b	8.02 ± 1.3a	1.97 ± 0.3c	1.44 ± 0.1c	2.46 ± 0.3cb	6.14 ± 1.1a
Octanal	0.26 ± 0.0c	0.65 ± 0.1b	0.58 ± 0.0b	0.59 ± 0.0b	2.29 ± 0.4a	0.51 ± 0.1b	0.41 ± 0.0b	0.79 ± 0.1b	2.03 ± 0.7a
*E*-2-Heptenal	0.20 ± 0.1f	0.71 ± 0.1e	0.59 ± 0.1e	0.90 ± 0.1e	4.68 ± 0.5b	2.22 ± 0.4d	2.58 ± 0.2d	3.59 ± 0.9c	10.15 ± 1.9a
Nonanal	0.87 ± 0.1c	1.69 ± 0.1b	2.35 ± 0.2b	5.22 ± 0.8b	11.34 ± 2.3a	1.01 ± 0.1bc	0.70 ± 0.2c	2.09 ± 0.6b	1.98 ± 0.5b
*E*-2-Octenal	0.11 ± 0.0d	0.83 ± 0.1c	0.54 ± 0.1c	0.92 ± 0.1c	6.51 ± 1.2a	1.24 ± 0.2bc	2.17 ± 0.1b	2.65 ± 0.9b	9.18 ± 1.9a
Benzaldehyde	1.48 ± 0.1bc	0.89 ± 0.1d	1.57 ± 0.1b	1.47 ± 0.0bc	1.69 ± 0.1ba	0.87 ± 0.1d	1.13 ± 0.0bc	1.99 ± 0.4a	1.25 ± 0.2bc
*E-E*-2,4-Nonadienal	0.02 ± 0.0d	0.27 ± 0.0c	0.15 ± 0.0c	0.51 ± 0.0b	1.47 ± 0.5a	0.09 ± 0.0d	0.08 ± 0.0d	0.25 ± 0.1cb	0.58 ± 0.2b
1-penten-3-ol	3.34 ± 0.0f	15.56 ± 2.1d	9.76 ± 0.7de	5.65 ± 0.3e	38.52 ± 2.9c	45.36 ± 5.6b	35.43 ± 1.4c	52.23 ± 6.1b	110.06 ± 12.4a
1-pentanol	1.43 ± 0.2f	47.88 ± 5.9c	25.18 ± 0.5e	33.54 ± 1.9d	122.76 ± 16.9a	33.95 ± 4.2d	25.58 ± 1.4e	33.94 ± 3.1d	92.28 ± 11.5b
*Z*-2-Penten-1-ol	0.19 ± 0.0d	0.45 ± 0.1c	0.39 ± 0.0c	0.56 ± 0.0c	1.70 ± 0.0b	0.68 ± 0.1c	0.52 ± 0.1c	1.18 ± 0.3b	2.09 ± 0.3a
1-hexanol	6.82 ± 2.5f	186.41 ± 23.0c	139.10 ± 3.8c	256.05 ± 36.5b	424.06 ± 74.31a	13.34 ± 1.3ef	7.94 ± 2.1f	17.89 ± 4.3e	34.84 ± 1.3d
1-octen-3-ol	0.76 ± 0.0d	9.59 ± 1.2c	6.28 ± 0.5c	7.23 ± 0.2c	31.06 ± 4.2a	5.15 ± 0.7c	4.95 ± 0.5c	7.87 ± 1.7c	20.99 ± 3.6b
Ethyl acetate	607.90 ± 20.6c	442.25 ± 50.2d	701.83 ± 10.2b	406.6 ± 22.5d	147.32 ± 13.8e	1037.14 ± 67.9a	1054.44 ± 48.4a	369.97 ± 65.5d	407.47 ± 32.6c
2-pentylfuran	1.44 ± 0.3d	3.77 ± 0.7c	5.14 ± 0.1c	9.19 ± 0.6b	24.11 ± 5.0a	2.86 ± 0.3c	4.35 ± 0.4c	10.79 ± 1.9b	8.63 ± 2.2b
1-octen-3-one	231.19 ± 57.9b	46.35 ± 11.1e	206.15 ± 98.9b	203.19 ± 45.1b	149.36 ± 22.5c	102.36 ± 67.3d	145.76 ± 48.1c	498.15 ± 125.5a	138.59 ± 53.2c
2-heptanone	0.62 ± 0.1d	2.05 ± 0.3c	1.51 ± 0.0c	2.45 ± 0.3c	8.31 ± 1.3a	1.99 ± 0.3c	1.82 ± 0.1c	13.01 ± 1.8b	1.43 ± 0.3c
6-methyl-5-hepten-2-one	0.42 ± 0.0e	0.93 ± 0.2c	1.09 ± 0.0bc	1.27 ± 0.2b	1.75 ± 0.2a	0.59 ± 0.1de	0.48 ± 0.0de	0.96 ± 0.3c	0.98 ± 0.1c
Propanoic acid	0.59 ± 0.2ab	0.22 ± 0.0b	0.71 ± 0.3a	0.92 ± 0.5a	0.55 ± 0.2a	0.41 ± 0.2ab	0.19 ± 0.1b	0.29 ± 0.1b	0.43 ± 0.2ab
Hexanoic acid	0.38 ± 0.1g	5.53 ± 0.8f	4.28 ± 0.8f	9.12 ± 0.5e	31.70 ± 10.4b	15.19 ± 2.4de	21.54 ± 2.7cd	41.06 ± 14.5c	106.92 ± 29.5a
**Sum**	**882.51 ± 74.1c**	**1577.28 ± 222.5b**	**1367.37 ± 99.5b**	**1406.94 ± 110.8b**	**2726.66 ± 593.9a**	**1603.04 ± 194.4b**	**1517.82 ± 108.5b**	**1736.19 ± 197.1b**	**2202.72 ± 359.3a**

Data are presented as mean ± SD of three independent experiments. Different letters within a row indicate significant differences (*p* < 0.05) for each cultivar.

**Table 6:** Volatile changes in Howard oil during storage.

Volatile Compound (mg/kg)		5 °C				23 °C			
Month	0	1	2	3	4	1	2	3	4
Propanol	10.44 ± 1.2f	6.99 ± 0.52f	7.00 ± 0.2f	24.04 ± 0.62e	122.14 ± 12.2ab	107.78 ± 4.5b	63.70 ± 9.02d	90.48 ± 1.3c	145.07 ± 19.6a
Butanal	10.16 ± 8.1b	23.64 ± 0.8a	0.53 ± 0.1d	1.80 ± 0.03cd	4.59 ± 0.4c	4.36 ± 0.4c	2.14 ± 0.1c	3.73 ± 0.4c	2.80 ± 0.9c
Pentanal	5.00 ± 0.2fe	3.33 ± 0.5e	2.99 ± 0.4e	8.03 ± 0.2df	25.22 ± 1.8c	51.19 ± 1.7a	24.06 ± 2.1c	43.43 ± 0.7b	28.53 ± 4.9c
Hexanal	87.99 ± 1.9f	117.01 ± 3.3e	108.86 ± 0.9e	304.92 ± 49.6d	993.69 ± 42.4a	705.27 ± 17.5b	378.46 ± 40.6d	675.37 ± 61.3b	591.17 ± 86.4c
*E*-2-Pentenal	0.43 ± 0.02f	0.37 ± 0.06f	0.20 ± 0.02f	0.90 ± 0.2e	1.01 ± 0.0e	2.77 ± 0.01c	1.98 ± 0.3d	3.15 ± 0.2b	6.21 ± 0.4a
Heptanal	0.89 ± 0.1c	1.26 ± 0.1c	1.33 ± 0.0c	2.53 ± 0.0b	6.75 ± 0.4a	3.05 ± 0.2b	1.52 ± 0.1c	3.11 ± 0.1b	3.13 ± 0.5b
*E*-2-Hexenal	0.11 ± 0.0f	0.52 ± 0.1ef	0.95 ± 0.0e	2.39 ± 0.1b	6.03 ± 0.4a	1.73 ± 0.0c	0.85 ± 0.1e	1.49 ± 0.0cd	1.29 ± 0.2d
Octanal	0.36 ± 0.0c	0.59 ± 0.0b	0.51 ± 0.1b	0.56 ± 0.1b	1.18 ± 0.1a	0.96 ± 0.1b	0.38 ± 0.0c	0.68 ± 0.1b	0.63 ± 0.1b
*E*-2-Heptenal	0.38 ± 0.0f	0.60 ± 0.1ef	0.55 ± 0.1f	1.07 ± 0.0e	2.94 ± 0.0d	5.74 ± 0.3a	3.65 ± 0.4c	5.34 ± 0.1a	4.61 ± 0.7b
Nonanal	4.19 ± 2.9cbe	3.71 ± 0.7d	3.95 ± 0.5d	9.11 ± 0.5b	19.02 ± 1.4a	4.03 ± 0.9c	2.03 ± 0.4e	2.21 ± 0.2e	3.53 ± 0.6c
*E*-2-Octenal	0.18 ± 0.0e	0.42 ± 0.0e	0.27 ± 0.0e	0.89 ± 0.0d	2.57 ± 0.1b	3.75 ± 0.1a	1.37 ± 0.3c	2.96 ± 0.0b	2.33 ± 0.3b
Benzaldehyde	1.53 ± 0.1cbd	1.18 ± 0.0e	1.19 ± 0.0e	1.36 ± 0.1de	1.55 ± 0.1cd	1.65 ± 0.1bc	1.94 ± 0.3abc	1.89 ± 0.2abc	1.97 ± 0.2a
1-pentanol	3.39 ± 0.1f	12.18 ± 0.3e	11.23 ± 0.0e	20.01 ± 0.3c	35.48 ± 1.5ab	39.79 ± 0.9a	16.66 ± 1.7d	34.89 ± 0.6b	21.12 ± 3.3c
*Z*-2-Penten-1-ol	0.13 ± 0.0d	0.29 ± 0.0d	0.36 ± 0.0d	0.67 ± 0.0c	1.51 ± 0.2a	1.22 ± 0.1b	0.69 ± 0.1c	1.19 ± 0.1b	1.20 ± 0.2b
1-hexanol	4.03 ± 0.0g	93.83 ± 2.6d	119.89 ± 2.5c	129.73 ± 1.4a	113.45 ± 4.4b	13.29 ± 0.3f	11.74 ± 1.4f	19.95 ± 0.2e	13.83 ± 2.1f
1-octen-3-ol	0.51 ± 0.0f	4.03 ± 0.3e	4.36 ± 0.1e	8.78 ± 0.6d	17.10 ± 0.6a	11.02 ± 0.5b	6.29 ± 1.1d	10.39 ± 0.5bc	9.34 ± 1.4c
3-carene	4.43 ± 0.2c	5.28 ± 0.1a	4.27 ± 0.1c	4.77 ± 0.1a	4.23 ± 0.2c	4.95 ± 0.3ab	4.42 ± 0.6bc	4.66 ± 0.2ab	3.48 ± 0.8b
2-pentylfuran	1.57 ± 0.0f	3.68 ± 0.3e	3.79 ± 0.0e	5.32 ± 0.3d	9.27 ± 0.5a	7.75 ± 0.4b	3.73 ± 0.4e	6.56 ± 0.1c	6.09 ± 0.9c
1-octen-3-one	479.07 ± 131.0ad	451.29 ± 81.5ad	545.08 ± 57.6a	243.90 ± 228.2dc	422.73 ± 43.0c	473.37 ± 61.9abd	457.52 ± 113.8abd	364.89 ± 100.2b	364.62 ± 131.8b
2-heptanone	1.03 ± 0.0e	1.88 ± 0.0d	1.59 ± 0.0d	2.67 ± 0.5c	4.39 ± 0.2a	3.60 ± 0.1b	1.92 ± 0.2d	3.32 ± 0.1b	2.48 ± 0.6c
6-methyl-5-hepten-2-one	0.30 ± 0.0e	0.93 ± 0.1c	1.21 ± 0.0b	1.74 ± 0.1a	1.84 ± 0.1a	0.69 ± 0.0d	0.77 ± 0.1d	0.99 ± 0.1c	0.83 ± 0.2d
Pentanoic acid	6.43 ± 0.7d	5.73 ± 0.3d	4.30 ± 0.6d	8.07 ± 0.8d	14.29 ± 1.4c	37.81 ± 2.5b	16.45 ± 1.0c	43.17 ± 3.3a	44.29 ± 9.3a
**Sum**	**876.69 ± 459.8c**	**1013.53 ± 548.6b**	**1118.79 ± 562.1b**	**1136.04 ± 524.1b**	**1810.99a**	**1998.57 ± 925.9a**	**1388.68 ± 790.3bc**	**1806.32 ± 881.9a**	**2446.59 ± 1123.8a**

Data are presented as mean ± SD of three independent experiments. Different letters within a row indicate significant differences (*p* < 0.05) for each cultivar.

**Table 7:** Volatile changes in Chandler oil during storage.

Volatile Compound (mg/kg)	5 °C					23 °C			
Month	0	1	2	3	4	1	2	3	4
Propanol	9.74 ± 1.5e	6.09 ± 1.3e	7.44 ± 0.8e	76.05 ± 21.6c	186.33 ± 89.3a	39.86 ± 1.1d	106.24 ± 0.6b	93.23 ± 12.4bac	165.00 ± 6.0b
Butanal	0.83 ± 0.1d	12.68 ± 0.2a	0.45 ± 0.1d	2.98 ± 1.0c	6.12 ± 2.7b	2.97 ± 0.3c	3.89 ± 0.4c	4.58 ± 0.7c	4.22 ± 0.3c
Pentanal	6.43 ± 1.9e	2.76 ± 0.1f	2.91 ± 0.1f	19.79 ± 0.6d	28.18 ± 14.9bd	38.31 ± 0.5c	47.61 ± 0.9ab	55.68 ± 5.7a	36.52 ± 0.3c
Hexanal	119.88 ± 15.9b	109.09 ± 2.1b	178.17 ± 0.2b	740.70 ± 356.7a	1297.99 ± 699.5a	598.03 ± 16.3a	714.94 ± 11.8a	954.13 ± 52.5a	833.29 ± 118.9a
*E*-2-Pentenal	0.39 ± 0.1d	0.21 ± 0.1d	0.16 ± 0.0d	1.55 ± 0.3c	1.30 ± 0.8c	1.37 ± 0.1c	2.33 ± 0.0bc	3.02 ± 0.2ab	3.85 ± 1.9a
Heptanal	1.06 ± 0.2e	1.60 ± 0.0d	1.80 ± 0.1d	6.07 ± 4.3bd	10.57 ± 5.8a	2.71 ± 0.0cd	2.89 ± 0.1c	4.51 ± 0.6c	4.13 ± 0.2c
*E*-2-Hexenal	0.32 ± 0.0c	0.68 ± 0.0b	1.62 ± 0.1b	5.48 ± 4.4a	9.01 ± 5.0a	1.31 ± 0.1b	1.43 ± 0.1b	2.05 ± 0.1b	1.64 ± 0.1b
Octanal	0.42 ± 0.0c	0.77 ± 0.0b	0.56 ± 0.1b	1.05 ± 0.6ab	1.51 ± 0.7a	0.76 ± 0.1b	0.86 ± 0.1b	1.00 ± 0.1b	0.81 ± 0.1b
*E*-2-Heptenal	0.32 ± 0.0f	0.54 ± 0.0e	0.92 ± 0.1d	3.13 ± 0.1c	3.42 ± 1.8c	3.47 ± 0.1c	5.96 ± 0.6a	6.28 ± 0.8a	4.91 ± 0.1b
Nonanal	2.09 ± 0.5e	6.51 ± 1.4c	6.37 ± 0.6c	19.93 ± 0.9b	27.17 ± 14.5a	3.15 ± 0.4e	2.46 ± 0.7e	4.86 ± 0.7cd	6.30 ± 1.2c
*E*-2-Octenal	0.26 ± 0.0c	0.42 ± 0.0c	0.47 ± 0.0c	1.43 ± 0.3c	2.53 ± 1.4ab	2.35 ± 0.0b	2.77 ± 0.3b	3.94 ± 0.5a	3.03 ± 0.1b
Benzaldehyde	0.99 ± 0.0c	1.28 ± 0.1bc	1.37 ± 0.1bc	1.96 ± 0.3ab	2.49 ± 1.3a	1.33 ± 0.1abc	1.56 ± 0.1ab	1.68 ± 0.3a	1.87 ± 0.1a
1-pentanol	3.29 ± 0.4e	9.15 ± 0.1d	7.93 ± 0.2d	22.39 ± 7.9c	39.65 ± 20.9b	31.69 ± 0.8bc	38.32 ± 0.7b	52.96 ± 4.7a	33.12 ± 0.2bc
*Z*-2-Penten-1-ol	0.10 ± 0.0b	0.24 ± 0.0b	0.38 ± 0.0b	0.99 ± 0.4a	1.79 ± 1.1a	0.67 ± 0.1a	1.08 ± 0.1a	1.21 ± 0.2a	1.41 ± 0.2a
1-hexanol	7.66 ± 0.9d	89.89 ± 1.0a	99.79 ± 0.9a	115.44 ± 5.1a	113.59 ± 64.2a	31.09 ± 1.4b	14.61 ± 0.7c	39.29 ± 3.5b	19.45 ± 0.7c
1-octen-3-ol	0.56 ± 0.1c	3.42 ± 0.2b	4.67 ± 0.1b	11.84 ± 5.9a	19.48 ± 11.3a	8.71 ± 0.2a	10.93 ± 0.6a	13.71 ± 1.5a	10.50 ± 0.2a
3-carene	2.44 ± 0.4ab	4.43 ± 0.0a	3.58 ± 0.0a	3.45 ± 0.2a	4.06 ± 2.3a	3.25 ± 0.1a	2.15 ± 0.1ab	2.31 ± 0.2ab	1.65 ± 0.1b
2-pentylfuran	1.41 ± 0.2c	4.32 ± 0.1b	3.81 ± 0.1b	7.47 ± 4.0a	11.12 ± 6.4a	5.15 ± 0.2ab	5.09 ± 0.2ab	8.47 ± 0.8a	7.00 ± 0.3a
1-octen-3-one	272.87 ± 7.7c	433.28 ± 115.4b	654.95 ± 63.8a	447.03 ± 46.9b	669.38 ± 278.7a	456.13 ± 41.3b	483.27 ± 100.9b	409.22 ± 38.6b	394.89 ± 68.9b
2-heptanone	1.03 ± 0.2c	2.01 ± 0.1bc	1.34 ± 0.1bc	2.47 ± 0.9b	4.82 ± 2.7a	2.76 ± 0.1b	3.11 ± 0.1ab	4.55 ± 0.4a	3.30 ± 0.1ab
6-methyl-5-hepten-2-one	0.24 ± 0.0c	0.91 ± 0.0b	1.15 ± 0.0b	1.59 ± 0.8ab	2.44 ± 1.3a	0.86 ± 0.1b	0.92 ± 0.1b	1.27 ± 0.1ab	0.99 ± 0.1b
Pentanoic acid	7.49 ± 1.1e	4.58 ± 0.4e	5.73 ± 1.5e	14.91 ± 1.7d	17.04 ± 6.2cd	23.03 ± 1.7c	41.80 ± 3.8b	63.79 ± 14.4a	54.66 ± 2.5a
**Sum**	**594.06 ± 236.7c**	**965.68 ± 528.1bc**	**1334.75 ± 646.8b**	**2159.77 ± 737.1b**	**3683.16 ± 1891.3a**	**1692.04 ± 786.3ab**	**2018.10 ± 909.0b**	**2329.37 ± 1002.9ab**	**2171.88 ± 1124.3ab**

Data are presented as mean ± SD of three independent experiments. Different letters within a row indicate significant differences (*p* < 0.05) for each cultivar.

## Data Availability

The data presented in this study are available on request from the corresponding author.

## Supplementary Materials

The following supporting information can be downloaded at: https://www.mdpi.com/article/10.3390/foods11193151/s1, Sensory attribute data and correlations between volatiles and sensory attributes can be found in the supplementary materials published online alongside the manuscript. Table S1: Changes in kernel sensory attributes during storage; Table S2: Changes in oil sensory properties during storage; Table S3: Correlations between evolution of volatile compounds and sensory attributes of kernels during storage; Table S4: Correlations between evolution of volatile compounds and sensory attributes of oil during storage.
