# Supplementary material for: Oxidative Stability of Walnut Kernel and Oil: Chemical Compositions and Sensory Aroma Compounds
Source: Foods. 2022 Oct 10;11(19):3151. doi: 10.3390/foods11193151 (PMC9563931; doi:10.3390/foods11193151)
Supplement: Supplementary file 1 [file foods-11-03151-s001.zip › foods-1946541-supplementary.pdf]

Supplementary Materials

Oxidative Stability of Walnut Kernel and Oil: Chemical Compositions and Sensory Aroma Compounds

Josephine Ampofo [1], Filipa S. Grilo [1], Sue Langstaff [2] and Selina C. Wang [1,*]

[1] Department of Food Science and Technology, University of California Davis, Davis, CA 95616, USA
[2] Applied Sensory, Fairfield, CA 94534, USA
* Correspondence: author: scwang@ucdavis.edu

Table S1. Changes in kernel sensory properties during storage.

Cultivar	Temp. (C)	Storage time (Month)	Sensory Attributes						
			Honey aroma	Cardboard aroma	Rancid aroma	Crunchy	Rancid flavor	Bitterness	Astringency/ Drying
Howard (H)									
		0	4.16a	0.89d	0.39d	3.55a	0.34c	2.06b	2.76bc
	5 °C	1	3.70a	0.76d	0.34d	3.88a	0.31c	2.18b	2.65c
		2	2.00b	3.38abc	1.36cd	2.19b	2.04ab	2.93ab	3.73ab
		3	1.59bc	3.98ab	2.04bc	2.25b	2.31ab	2.99ab	3.66abc
		4	0.86bc	4.59a	2.01bc	1.91b	3.29a	3.75a	4.16a
	23 °C	1	0.53c	2.78bc	4.65a	3.48a	3.35a	3.61a	4.03a
		2	1.85b	1.44d	1.16cd	3.04ab	1.53bc	3.36a	3.51abc
		3	0.43c	3.01bc	4.54a	3.66a	3.06a	3.51a	3.98a
		4	1.04bc	2.68c	3.10b	4.06a	2.19ab	2.98ab	3.49abc
Chandler (C)									
		0	4.48a	0.63d	0.35e	3.28abcd	0.38e	1.64c	2.41a
	5 °C	1	3.09b	1.26cd	0.93e	3.85ab	0.95de	2.51bc	2.86a
		2	2.22bc	3.18ab	1.50de	2.50d	1.43cde	2.98ab	2.76a
		3	2.19bc	3.83a	1.18de	2.90bcd	1.84bcd	2.90ab	2.96a
		4	1.55cd	4.38a	2.64cd	2.53cd	2.83ab	3.28ab	3.05a

23 °C	1	0.78d	3.01ab	4.31ab	3.71abc	3.40a	2.59bc	3.14a
	2	0.93d	2.26bc	3.23bc	3.58abcd	2.80ab	2.80ab	3.65a
	3	0.69d	3.30ab	4.76a	3.94ab	3.71a	3.31ab	3.50a
	4	1.13cd	3.13ab	3.15bc	4.29a	2.66abc	3.65a	3.30a

Different letters within a column indicate significant differences ($P < .05$) among temperature treatments for each cultivar.

Table S2. Changes in oil sensory properties during storage

Cultivar	Temp. (C)	Storage time (Month)	Sensory Attributes												
			Overall aroma intensity	Buttery aroma	Fresh nuts aroma	Toasted/ burnt aroma	Grainy aroma	Cardboard/ woody aroma	Rancid aroma	Overall flavor intensity	Fresh nuts flavor	Grainy flavor	Cardboard flavor	Rancid flavor	Greasy/ thick texture
Howard															
		0	3.55cd	2.15ab	1.69abc	0.50a	0.44f	0.36d	0.96c	3.20c	1.90a	0.78c	1.00c	0.66b	1.43c
	5 °C	1	4.76bc	1.11bc	2.61a	2.26a	2.68bc	2.06abc	0.31c	4.53b	2.36a	2.80ab	1.91bc	0.38b	1.66c
		2	5.63ab	1.31bc	1.68abc	1.44 a	4.10a	2.50abc	0.50c	5.19ab	1.93a	4.13a	3.08ab	0.91b	2.01bc
		3	4.49bcd	1.26bc	1.61abc	1.28a	2.40bcd	3.53a	0.56c	4.84ab	2.22a	2.75ab	3.69a	0.64b	1.65c
		4	6.36a	1.25bc	1.89ab	2.48 a	3.58ab	2.96ab	0.96c	5.69a	1.73a	3.76a	3.49a	1.73b	2.46abc
	23 °C	1	3.39d	1.55bc	1.25bc	1.86a	1.21def	0.96cd	1.09bc	4.10bc	1.21a	1.35bc	1.55c	1.23b	2.13bc
		2	3.76cd	2.83a	0.80c	1.71a	0.99ef	1.09cd	0.76c	4.16bc	1.19q	0.86c	2.05bc	1.33b	2.01bc
		3	4.38bcd	1.45bc	1.09bc	1.66 a	2.10cde	2.08abc	2.49a	4.90ab	0.75a	1.80bc	2.36abc	3.39a	2.86ab
		4	4.65bcd	0.90c	1.11bc	1.80a	1.68cdef	1.76bcd	2.31ab	5.16ab	1.09a	1.91bc	1.98bc	3.28a	3.43a
Chandler															
		0	3.41a	2.18a	0.83a	1.45a	1.18c	0.79b	0.59a	3.79a	1.31a	1.13b	1.30a	1.34a	1.25a
	5 °C	1	5.03a	1.29a	1.89a	1.93a	1.50c	3.23a	2.00a	5.21a	2.16a	2.14b	2.29a	2.80a	2.29a
		2	4.96a	1.21a	2.08a	1.73a	4.68a	1.91ab	0.56a	4.86a	2.19a	4.55a	2.06a	0.54a	1.08a
		3	4.39a	1.56a	1.63a	2.76a	2.34bc	2.16ab	0.71a	3.74a	1.58a	2.18b	2.37a	1.36a	1.16a
		4	5.28a	1.19a	2.08a	2.20a	3.56ab	3.15a	0.76a	5.00a	1.96a	3.76a	3.20a	1.51a	1.73a

23 °C	1	3.45a	1.65a	2.16a	1.46a	1.45c	1.16b	0.90a	4.05a	1.89a	1.86b	1.68a	1.89a	1.99a
	2	4.48a	2.58a	1.14a	1.63a	1.68c	1.70b	1.68a	3.96a	1.89a	1.63b	2.23a	2.71a	2.60a
	3	4.83a	2.78a	1.80a	1.75a	1.64c	1.73b	2.49a	4.75a	1.48a	2.11b	2.23a	3.01a	1.95a
	4	4.39a	2.34a	0.95a	2.53a	1.54c	1.61b	1.55a	4.09a	1.36a	1.96b	1.44a	1.71a	2.34a

Different letters within a column indicate significant differences ($P < .05$) among temperature treatments for each cultivar.

Table S3. Correlations between evolution of volatile compounds and sensory attributes of kernels during storage

**Volatile**			5 °C					23 °C		
**Sensory attributes**	Bitterness	Astringency	Rancid aroma	Honey aroma	Cardboard aroma	Bitterness	Astringency	Rancid aroma	Honey aroma	Cardboard aroma
Butanal	-0.532*	-0.492	-0.515*	0.628*	-0.528*	-0.968**	-0.911**	-0.700**	0.906**	-0.708**
Pentanal	0.889**	0.805**	0.872**	-0.882**	0.846**	0.464	0.456	0.392	-0.600*	0.566*
Hexanal	0.918**	-.823**	0.843**	-0.874**	0.838**	0.371	0.402	0.418	-0.588*	0.613
Octanal	0.887**	0.771**	0.776**	-0.840**	0.779**	0.369	0.426	0.475	-0.596*	0.646**
Nonanal	0.933**	0.865**	0.903**	-0.922**	0.893**	0.290	0.372	0.487	-0.589*	0.681**
Benzaldehyde	0.439	0.585*	0.578*	-0.582*	0.609*	0.232	0.222	0.204	-0.416	0.416
1-octen-3-one	0.058	0.100	0.191	-0.205	0.166	0.118	0.127	0.192	-0.361	0.383
Nonadienal	0.909**	0.805**	0.789**	-0.840**	0.800**	0.394	0.462	0.509	-0.599*	0.660**
1-hexanol	0.943**	0.921**	0.968**	-0.973**	0.958**	0.545*	0.576*	0.549*	-0.700**	0.694**
Propanol	0.873**	0.762**	0.776**	-0.821**	0.773**	0.199	0.218	0.268	-0.464	0.499
E-2-pentenal	0.939**	0.856**	0.866**	-0.915**	0.870**	0.258	0.287	0.337	-0.525*	0.563*
Heptanal	0.916**	0.819**	0.816**	-0.862**	0.822**	0.395	0.433	0.448	-0.602*	0.630*
E-2-hexenal	0.909**	0.831**	0.882**	-0.888**	0.862**	0.538*	0.552*	0.499	-0.666**	0.643**
E-2-heptenal	0.880**	0.774**	0.768**	-0.812**	0.773**	0.462	0.471	0.425	-0.602*	0.586*
E-2-octenal	0.867**	0.756**	0.714**	-0.773**	0.735**	0.463	0.507	0.495	-0.597*	0.616*
1-pentanol	0.932**	0.877**	0.933**	-0.936**	0.914**	0.501	0.519*	0.478	-0.640*	0.627*
Z-2-penten-1-ol	0.922**	0.819**	0.770**	-0.843**	0.798**	0.201	0.250	0.342	-0.499	0.568*
1-octen-3-ol	0.950**	0.866**	0.852**	-0.911**	0.867**	0.493	0.509	0.456	-0.605*	0.588*
2-pentylfuran	0.913**	0.851**	0.889**	-0.905**	0.879**	0.455	0.489	0.483	-0.633*	0.646**

6-methyl-5-hepten-2-one	0.704**	0.741**	0.792**	-0.826**	0.793**	0.482	0.446	0.346	-0.601*	0.531*
Propanoic acid	0.020	0.037	-0.017	-0.092	0.028	0.033	0.107	0.209	-0.096	0.167

Significant difference at $P < .05$ is presented as *, whereas significant difference at $P < .01$ is presented as **.

Table S4. Correlations between evolution of volatile compounds and sensory attributes of oil during storage

**Volatile**	**5 °C**				**23 °C**			
**Sensory attributes**	Rancid flavor	Rancid aroma	Buttery aroma	Overall aroma intensity	Rancid flavour	Rancid aroma	Buttery aroma	Overall aroma intensity
Hexanal	0.922**	0.920**	-0.422	0.507	0.717**	0.845**	0.498	0.841**
Pentanal	0.002	-0.289	-0.174	0.222	0.881**	0.849**	0.452	0.776**
Nonanal	0.003	-0.241	-0.311	0.357	0.721**	0.864**	0.477	0.834**
Octanal	0.183	0.009	-0.396	0.444	0.829**	0.865**	0.481	0.803**
E-2-heptenal	0.077	-0.115	-0.419	0.467	0.115	0.511	0.263	0.534*
Pentanoic acid	0.015	-0.144	-0.403	0.431	0.469	0.709**	0.458	0.769**
1-octen-3-one	0.075	0.168	-0.744**	0.737**	0.587*	0.616*	0.087	0.394
1-hexanol	-0.223	-0.135	-0.692**	0.674**	0.529*	0.369	0.037	0.347
Propanol	0.052	-0.220	-0.320	0.384	0.587*	0.616*	0.087	0.394
E-2-pentenal	0.023	-0.281	-0.070	0.086	0.331	0.618*	0.491	0.762**
Heptanal	0.034	-0.190	-0.331	0.377	0.395	0.662**	0.463	0.726**

E-2-hexenal	-0.027	-0.233	-0.341	0.375	0.617*	0.853**	0.485	0.819**
E-2-heptenal	-0.062	-0.269	-0.334	0.348	0.754**	0.884**	0.468	0.822**
E-octenal	0.038	-0.193	-0.360	0.408	0.839**	0.878**	0.579*	0.875**
1-pentanol	0.089	-0.129	-0.409	0.459	0.771**	0.881**	0.480	0.825**
Z-2-penten-1-ol	0.004	-0.200	-0.379	0.420	0.871**	0.897**	0.494	0.812**
1-octen-3-ol	0.014	-0.175	-0.420	0.455	0.557*	0.768**	0.498	0.824**
2-pentylfuran	0.105	-0.053	-0.454	0.491	0.805**	0.868**	0.468	0.820**
2-heptanone	0.205	0.003	-0.412	0.478	0.673**	0.865**	0.452	0.799**
6-methyl-5-hepten-2-one	0.008	-0.103	-0.542*	0.564	0.807**	0.918**	0.532*	0.839**
Pentanoic acid	-0.108	-0.334	-0.022	0.033	0.766**	0.870**	0.444	0.801**

Significant difference at $P < .05$ is presented as *, whereas significant difference at $P < .01$ is presented as **.
